# Personal networks: a tool for gaining insight into the transmission of knowledge about food and medicinal plants among Tyrolean (Austrian) migrants in Australia, Brazil and Peru

**DOI:** 10.1186/1746-4269-10-1

**Published:** 2014-01-07

**Authors:** Ruth Haselmair, Heidemarie Pirker, Elisabeth Kuhn, Christian R Vogl

**Affiliations:** 1 Department for Sustainable Agricultural Systems, Knowledge Systems and Innovations, Division of Organic Farming, University of Natural Resources and Life Sciences, Vienna, Austria

**Keywords:** Social network analysis, Personal networks, Knowledge transmission, Food knowledge, Medicinal plant knowledge, Migration, Knowledge traditions

## Abstract

**Background:**

Investigations into knowledge about food and medicinal plants in a certain geographic area or within a specific group are an important element of ethnobotanical research. This knowledge is context specific and dynamic due to changing ecological, social and economic circumstances. Migration processes affect food habits and the knowledge and use of medicinal plants as a result of adaptations that have to be made to new surroundings and changing environments. This study analyses and compares the different dynamics in the transmission of knowledge about food and medicinal plants among Tyrolean migrants in Australia, Brazil and Peru.

**Methods:**

A social network approach was used to collect data on personal networks of knowledge about food and medicinal plants among Tyroleans who have migrated to Australia, Brazil and Peru and their descendants. A statistical analysis of the personal network maps and a qualitative analysis of the narratives were combined to provide insight into the process of transmitting knowledge about food and medicinal plants.

**Results:**

56 personal networks were identified in all (food: 30; medicinal plants: 26) across all the field sites studied here. In both sets of networks, the main source of knowledge is individual people (food: 71%; medicinal plants: 68%). The other sources mentioned are print and audiovisual media, organisations and institutions. Personal networks of food knowledge are larger than personal networks of medicinal plant knowledge in all areas of investigation. Relatives play a major role as transmitters of knowledge in both domains.

**Conclusions:**

Human sources, especially relatives, play an important role in knowledge transmission in both domains. Reference was made to other sources as well, such as books, television, the internet, schools and restaurants. By taking a personal network approach, this study reveals the mode of transmission of knowledge about food and medicinal plants within a migrational context.

## Background

Knowledge about food and medicinal plants is not only based on an individual’s experience, but is gained from other close contacts through learning processes and therefore reflects prevailing social conditions [1]. In ethnobotanical studies, specific knowledge transmitted within a group from one generation to another and/or to another locality is often referred to as “traditional knowledge”. The use of the term “traditional knowledge” has recently been questioned by some authors within the ethnobotanical community as the term “traditional” is too vague and misleading in this context. However, human cultures are continually evolving through transgenerational transmission (cultural and historical continuity), new interpretations of rediscovered knowledge or the acquisition of new knowledge. These processes tend to be experimental, dynamic and closely related to a way of life in a particular geographic area [2]. Therefore, within this study the terms “food knowledge” and “medicinal plant knowledge” are used.

According to the social anthropologist Fredrik Barth, knowledge is anything “a person employs to interpret and act on the world” [3]. It includes “feelings (attitudes), information, embodied skills, verbal taxonomies and concepts” [3]. Knowledge is the basis for communication in human relationships. It is embedded in a specific social context and acquired during different phases of socialisation. Barth [4] identified three main interconnected aspects for the analysis of “traditions of knowledge”: i) the content of knowledge – a “corpus of assertions and ideas of the world”, which ii) “is communicated by one or several media in the form of words, symbols, gestures or actions”, and iii) is “distributed, communicated, employed and transmitted within a series of instituted social relations” [4]. This framework for defining “knowledge” and analysing “traditions of knowledge” forms the basis for this study since it emphasises not only the content of knowledge, but its constitution, transmission and distribution as well.

Knowledge about cultural or societal practices is primarily transmitted in a non-verbal way, *i.e.* “knowledge transmission tends to occur in the context of everyday activities through observation and” hands-on “ practice”. There is a minimum of direct and verbal instruction [5]. In day-to-day activities in particular, non-verbal knowledge is very pronounced. Borofsky [6] distinguishes between implicit and explicit knowledge. Humans internalise and embody implicit knowledge as so-called habitus and acquire explicit knowledge during their lifetime [7]. The implicit memory and storage of knowledge is interdependent with the collective memory that is formed by the history of a specific group. Knowledge can be shared by members of a group, but its distribution is influenced by factors such as sex, age, relationship and employment [8]. What knowledge is acquired by whom and at what moment and what knowledge is then subsequently passed on depends significantly on the respective person’s standing as well as on the social context [9], a group’s stability and life events, *i.e.* migration, language and the degree of exposure to the host culture [10,11]. New circumstances will influence the body of knowledge transmitted [12-21]. The recently-published study on the transformation of the traditional knowledge of medicinal plants among Tyroleans and Tyrolean migrants in Australia, Brazil and Peru shows that the continuation, substitution and replacement of medicinal plants are strategies that have taken place at differing rates among migrants, depending on local circumstances in the migrants’ areas [22].

Most authors of ethnobotanical research of knowledge transmission concerning food [10] or medicinal plants [23-27] refer to the model developed by Cavalli-Sforza and Feldmann [28], where cultural transmission has been defined as “a process of social reproduction in which a culture’s technological knowledge, behaviour patterns and cosmological beliefs are communicated and acquired” [29]. The transmission of culture is reliant on intergenerational processes. According to this model, three different modes of knowledge transmission can occur between individuals: i) from parent to offspring (vertical transmission), ii) between any two individuals of the same generation (horizontal transmission) and iii) from non-parental individuals of the parental generation to members of the filial generation (oblique transmission) which covers relationships of many to one (*i.e.* older and younger) and one to many (*i.e.* teacher-student relationships). In the model of Cavalli-Sforza and Feldmann [28] vertical transmission is seen as highly conservative and may maintain the status quo. Innovation at an individual level will be very slow to spread to others if there is no other type of knowledge transmission employed in the community. Both modes of vertical and horizontal transmission can favour a rapid spread to others and intracultural variation of knowledge can be high. Therefore, individual differences occur according to differences in individual experiences and knowledge. Within the path of one to many (oblique transmission), communication is highly efficient and therefore changes in knowledge may occur rapidly [28].

Previous studies of knowledge transmission that have been examined state that knowledge is transmitted in both illiterate and literate communities, predominantly orally and “vertically”, that is from parent to child [2,8,24,26,27,29], and that females contribute more to the transmission of knowledge about food and medicinal plants [8,24,30]. However, there are studies that contradict these findings. For example, among the Bolivian Tsimané oblique transmission is perceived to be more important, which may be due to a different social organisation [25].

So far, studies on knowledge transmission in ethnobotanical research have tended to concentrate on individuals as knowledge transmitters. There has not been much focus on the transmission of knowledge about food and medicinal plants through media such as the internet, books, journals and television. In a more globalised world in which individuals increasingly have access to modern media, even in remote parts of the world, there is a greater opportunity to record knowledge on audio or visual media and publish it on the internet. Therefore, these media need to be taken into consideration in future ethnobotanical research. Digital technology adds a new dimension to knowledge transmission and the number and diversity of technological platforms and applications will continue to grow [27].

Globalisation processes and the global environmental crisis affect local resource use strategies and the transmission and acquisition of knowledge in profound ways. Improved understanding of how people pass on knowledge offers important pointers for future biodiversity management practices and for strengthening the resilience of knowledge traditions by optimising learning environments and methods so that global changes and challenges are not seen merely as a threat. Reyes-Garcia [31] points out that in ethnobotanical research there is a lack of specifically quantitative data sets on the processes of knowledge transmission to allow an understanding of the different paths through which knowledge is transmitted. Particularly at a time of globalisation and change, future research on the transmission of local knowledge related to food and medicinal plants will be important for providing insight into the characteristics, processes and trends of ethnobotanical knowledge systems [26,27,32].

This study provides a quantitative and qualitative exploration of the transmission of knowledge about food and medicinal plants among Tyroleans who emigrated to Australia (from the 1950s), Brazil (between 1933 and 1938) and Peru (in 1859 and 1868). It focuses on the transmission of knowledge in particular, by detecting knowledge sources in the specific knowledge traditions and comparing the results from the three different study sites.

By using a social network approach, personal networks of informants were identified that consist of a variety of different sources of knowledge (*e.g.* family, friends, television, the internet *etc.*) that are consulted in order to learn about food practices and medicinal plants. The quantitative data collected from Tyrolean migrants and their descendants in three different parts of the world are presented and discussed with regard to: i) the content of their knowledge, ii) the sources of their knowledge about food and medicinal plants, iii) the characteristics of this knowledge and iv) the reasons and methods for learning.

### The content of knowledge about food and medicinal plants

Knowledge about food and medicinal plants encompasses knowledge about the production, preparation and consumption of food, drink and preserved food, and knowledge about the harvesting, processing, use and application of medicinal plants, as well as the derivation of plant-based resources and their regional and ethnic origin. Practical knowledge is of great importance in both domains since the preparation of recipes and the application of medicinal remedies require manual skills.

**Figure 1:**
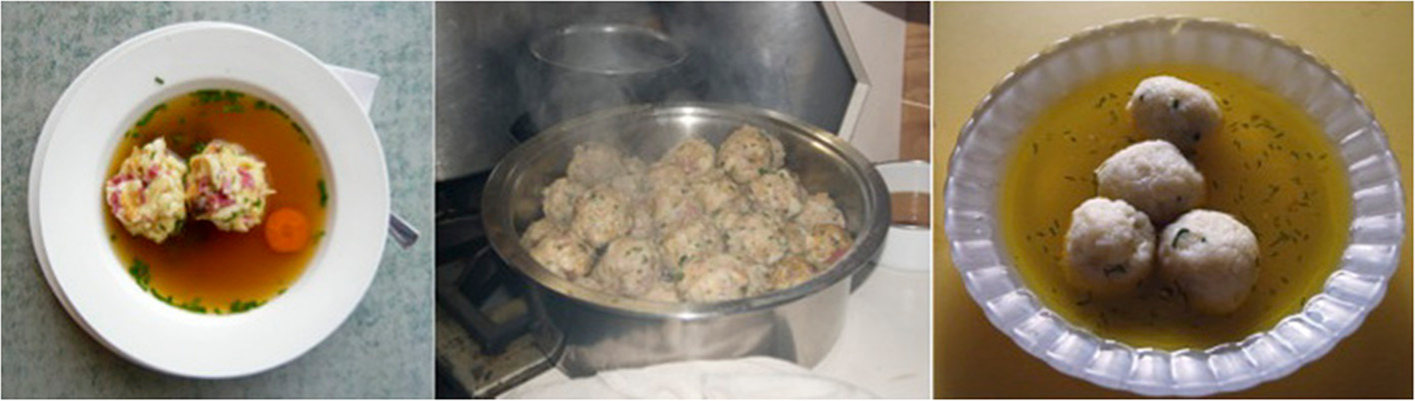
Tyrolean dumplings in Australia, Brazil and Peru (from left to right).

The food available to migrating Tyroleans upon their arrival in Australia, Brazil and Peru was substantially different from what they had known in Austria. Therefore their cultural, economic and social practices relating to the production and consumption of food were influenced by the food resources available locally and gradually changed over time. One example of is the dish *Tiroler Knödel (*Tyrolean dumplings) served in beef broth. It is an iconic Tyrolean dish that is known in each of the three research areas. The composition of the dumplings varies in the research areas according to the resources available. Figure 1 illustrates the variations within the three research sites. In Austrian-Tyrolean cuisine the dumplings are made from dry bread cubes (nowadays a commercial product), eggs, milk, onions, chives, sausages or smoked bacon, flour, salt and pepper [33]. The dumplings are then served in beef broth [33]. In Treze Tílias and Australia, dumplings are usually made in a similar way as in Tyrol, although the bread cubes are usually homemade in Brazil and Australia since they are not commercially available as they are in Tyrol. Therefore, according to the consistency of the bread, the amount of eggs, milk and flour has had to be adapted in order to reach the right consistency. In Australia and Brazil, boiled meat leftovers or smoked sausage like salami are often used if smoked bacon is not available. The use of salami in Tyrolean dumplings is not common in Tyrol, but is used in Treze Tílias as a substitute for other kinds of sausage that are not available. In Treze Tílias, where it is more common to use the generic term *Knödel* instead of *Tiroler Knödel*, they are a popular dish served in local restaurants and in the homes of migrants. Tyrolean migrants in Australia would sometimes prepare Tyrolean dumplings at home and dumplings occasionally appear on the menu in Austrian clubs.

In Pozuzo dumplings are called *Albóndigas de arroz.* Since there was no flour available when migrants arrived, bread cubes were substituted with rice, grated corn and grated manioc. Other ingredients of the “invented” rice dumplings are eggs, chives, garlic and salt. Nowadays some Pozuzians use semolina instead of the corn that used to be grated by hand, and wheat flour instead of manioc, also grated by hand, as both products are now available ready-made in grocery stores in Pozuzo. Depending on taste and custom, pieces of the chicken used for preparing the accompanying broth are added to the dumpling dough as well. The dumplings are served in chicken broth [34]. They are perceived to be a typical Pozuzian dish whose origin goes back to the knowledge on the preparation of “Tyrolean dumplings” by their Tyrolean ancestors. Although nowadays bread – essential to the making of Tyrolean dumplings – is available in Pozuzo, the dish is not offered in local restaurants or prepared in people’s homes.

As the content of medicinal plant knowledge among Tyrolean migrants in Australia, Brazil and Peru has already been the subject of a recently published work by the authors, only a short summary is given of the most important outcomes of this study. For detailed information about the content of knowledge of medicinal plants among Tyroleans and Tyrolean migrants and the transformation this knowledge has undergone during migration processes, please refer to the article published recently on this subject [22].

The use of traditional Tyrolean medicinal plants continued concurrently when Tyrolean migrants arrived in their new environments, and then progressively moved towards an adoption of the existing traditions of medicinal plant use in the host country. Some practices were abandoned and new elements integrated into the medicinal plant knowledge system of the migrants. The choice of medicinal plant use – decades after the migration of Tyroleans to Australia, Brazil and Peru – was heavily influenced by existing environmental conditions (accessibility of traditional Tyrolean plants) and social conditions in the country of arrival, the prevalent healthcare system, particular illnesses in the specific areas, opportunities for importing plants, and established networks with the local population as well as back in the home country. Use of cosmopolitan plants *e.g.* garlic (*Allium sativum* L.*)*, wormwood (*Artemisia absinthium* L*.*), cabbage (*Brassica oleracea* L*.)*, St. John’s Wort (*Hypericum perforatum* L*.)*, chamomile (*Matricaria chamomilla* L.), plantain (*Plantago* spp.) and stinging nettle (*Urtica* spp.) is in any case most likely to continue.

## Methods

The immigration countries for the research project were selected to represent: i) different social milieus and structures of settlement, ii) different economic conditions, iii) different environmental conditions, iv) different periods of immigration and v) sizeable Tyrolean emigrant populations. A brief history of migration to each area is presented in Table 1.

**Table 1:** Overview of the research areas

**Criteria**	**Peru**	**Brazil**	**Australia**
**Social milieu and structure of settlement**	municipality (7,760 inhabitants), no road access until 1975	municipality (6,400 inhabitants), good road access	Tyroleans dispersed in different urban and peri-urban areas
**Economic conditions**	high importance of agriculture and forestry work	agriculture, tourism, woodwork	high importance of non-agricultural labour
**Environmental conditions**	humid, tropical climate	temperate climatic conditions	ranges from temperate climate to subtropical climate
**Type of immigration**	colonisation project under governmental agreement	organised group funded by the Austrian government	individual immigration mostly under the Assisted Passage Scheme
**Date of immigration**	1859 and 1869: approx. 310 Tyroleans	1933 to 1939: approx. 560 Tyroleans	1952 to 1961: approx. 17,000 Austrians

### Ethnogeography and biogeography of research sites

#### ***Australia – New South Wales, Queensland, South Australia and Victoria***

After the Second World War, Austria’s weak economic situation provided a strong incentive for emigration. Many Austrian migrants wanted to improve their living standards, and between 1952 and 1961 almost 17,000 Austrians took advantage of the opportunity of the Assisted Passage Scheme (APS) [35]. The APS agreement was signed between the Australian Government and the Provisional Government in Austria in 1952 and allowed Austrians to apply for immigration. It is not clear how many Tyroleans have migrated since 1948 as no statistical records are kept for specific Austrian provinces (Bundesländer). However in 2008 the estimated resident population of Australians born in Austria was 20,828 [36]. The interviewees live in the greater metropolitan area of Sydney (33°53′S, 51°12 O) in New South Wales, on the Gold Coast (27°59′S, 153°22’O) in Queensland, in Adelaide (34°55′0″S, 138°36′0″E) in South Australia, in the greater metropolitan area of Melbourne (37°50′S, 145°00’O) in Victoria, and in Alice Springs (23° 42′ S, 133° 48’ O) in the Northern Territory. Austrian migrants in Australia have not formed separate communities and are mainly dispersed throughout Australia’s urban and peri-urban areas. Having learnt English, Austrian migrants became assimilated fairly quickly and participated in creating the identity of what is now a multicultural Australian society with numerous migrants from other European countries in the 1950s. Like other ethnic groups who migrated to Australia, Austrian migrants set up Austrian national clubs in the main cities where Austrian culture is still celebrated today, predominantly by older members, through various club activities. Many of the respondents regularly travel to Austria and maintain contact with their Austrian relatives and friends through the modern convenience of internet applications such as e-mail and Skype. Tyroleans in Australia merged into the melting pot of the considerable number of migrants from all over the world, hence modern Australian cuisine involves a blend of many different ethnic traditions. The migrants adopted many of the host country’s food habits and maintained the custom of occasionally preparing popular Tyrolean dishes, *i.e.* at family get-togethers. Immigrant Tyroleans, some of whom were chefs and quite inventive, opened up restaurants, bakeries and clubs where Tyrolean specialities such as *Apfelstrudel* (apple strudel), *Tiroler Knödel* (Tyrolean dumplings), *Leberknödel* (liver dumplings), *Schweinsbraten* (roast pork), *Brezen* (pretzels) or *Schnaps* (schnapps) are served.

#### ***Brazil – Treze Tílias***

Treze Tílias (27° 0′ 0″ S, 51° 24′ 0″ W) is a municipality in Santa Catarina covering an area of 185,205 km^2^ in southern Brazil (796 m above sea level) with a population of 6,341, of whom 4,715 live in urban areas and 1,626 in rural areas [37]. The population now mainly consists of descendants of people of Austrian, Italian and German origin and Brazilians. The landscape is characterised by hills and determined by temperate climatic conditions. Treze Tílias was founded by a group of Austrian migrants. Between September 1933 and January 1938, 789 Austrians – of whom 560 were Tyrolean – moved to Treze Tílias. Over 300 of them came from the Lower Inn Valley in Tyrol [38,39]. The migration project was initiated by a former Austrian Minister of Agriculture, Andreas Thaler, and was funded by the Austrian Government. The migration project was designed to allow émigré Austrians to maintain their customs and traditions. A location was chosen that was said to be free of other populations and a long way from the nearest city in order to avoid “cultural assimilation” [40]. When the migrants arrived in the Treze Tílias area, there was barely any infrastructure and migrants more or less had to build the settlement themselves. The outbreak of the Second World War halted immigration from Austria, but official contact with Austria was re-established when the war ended. Ethnic tourism was introduced in the 1970s, supported by the Tyrolean government, which led to improvements in the economic situation [39,41,42]. The population rose from the 1980s in particular, mainly due to people relocating from other parts of Santa Catarina (IBGE 2010). In 1991, a telenovela filmed in Treze Tílias and broadcast on public television across Brazil in 1991 made “Tyrolean Brazil” famous. Today, Treze Tílias is considered a tourist destination, predominantly for Brazilians, and Tyrolean dances, music and other traditions are performed for tourists.

After their arrival, the migrants’ food traditions were challenged by their new surroundings, and in the early years the life of the migrants was marked by a scarcity of food. Therefore the migrants had to live mainly from subsistence farming. Most of the staple foods to which the migrants were accustomed in Austria, such as wheat, rye and milk, were not available and were substituted by the staples of maize, manioc and sweet potato. The new circumstances forced the migrants to learn how to prepare what had until then been unknown food staples. Some Brazilian food traditions were welcomed in the cuisine of the migrant Austrians very early on, such as the South Brazilian *churrasco (spit roast)* which was prepared in the early years by the settlers themselves [39,43]. Nowadays, on Sundays and for festivities, families with Tyrolean backgrounds embrace the tradition of preparing c*hurrasco* at family gatherings and parties. Depending on the migrational background, personal taste and the food preferences of each family, Tyrolean food continued to be cooked in families’ homes, including old, simple dishes such as *Riebla* (a dish made of maize flour which was made a great deal when the immigrants first arrived and resources were scarce) or *Krapfen* (various kinds of doughnuts with sweet or sour fillings which are then fried or baked). However, families with a Tyrolean migration background also cook international food such as pasta and rice dishes as well as Brazilian dishes, with the Brazilian “national dish” consisting of rice and beans being particularly popular.

While most restaurants in Treze Tílias offer Brazilian and international food such as burgers, pizza and special Brazilian meat dishes, ethnic restaurants also serve a range of Austrian dishes to visitors and locals. This range of ethnic food includes *Knödel* (dumplings), *Gulasch* (goulash), *Schweinsbraten* (roast pork), *Sauerkraut* (fermented cabbage), *Würstel* (sausages), *Wiener Schnitzel* (Viennese Schnitzel) and *Apfelstrudel* (apple strudel).

#### ***Peru - Pozuzo***

Pozuzo (10° 4′ 0″ S, 75° 32′ 0″ W) is a district in the department of Oxapampa in the Central Andean region (750 m above sea level) of Pasco in Peru and has a tropical climate. Colonisation by migrants stems from an invitation from the Peruvian state to colonise the land leading up to the Amazon. The Peruvian state planned to connect the Pacific and Atlantic Oceans by a direct route. This plan was later dropped due to the construction of the Panama Canal [44]. Following a long and exhausting two-year journey from Europe, 170 Tyrolean and German immigrants founded the capital of Pozuzo (Pozuzo Centro) in 1859. A second group arrived in 1868. Due to difficult travelling conditions, in all only around 350 of the 629 Tyrolean and German emigrants who left Europe arrived in Pozuzo between 1859 and 1868. A road to Pozuzo was only built in 1974. Until then it was a three-day walk to the nearest towns. Due to this isolation the migrants mainly endured subsistence living. However, this contributed to the continuation of their customs and language [44-46]. Once the road was completed, migration from other parts of Peru increased. The district of Pozuzo now has a population of 7,760, of whom just 1,038 live in urban areas [47]. About one third of them are descendants of Tyrolean and German migrants [48]. Contact with Tyrol was re-established from the 1930s onwards and intensified from the 1970s, with ongoing financial assistance being provided by Tyrol and Germany [49]. Today, Pozuzians see themselves as Peruvians, but at the same time they emphasise their special Tyrolean-German heritage, which is also expressed in their food habits. In Tyrolean dishes such as *Apfelstrudel* (apple strudel), Pozuzians replaced apples – not available in Pozuzo when they arrived – with bananas. Another example for their innovative adaptations is the preparation of *Knödel* (dumplings) with rice instead of white bread. In addition to Tyrolean cuisine, local dishes of Peruvian origin such as *pachamanca* are part of Pozuzian cuisine nowadays. Pozuzian cuisine contains elements of Peruvian food and locally transformed food of Tyrolean and German origin. Some of the Peruvian dishes were integrated into Pozuzian cuisine a long time ago; others have only been known for a short time and are mainly prepared to add variety to day-to-day meals. The 150th anniversary of the foundation of the colony was commemorated in celebrations in 2007 in Tyrol and in 2009 in Pozuzo. These events have led to a revival in Austrian-German food heritage, which is not only represented by continuously passing on local food traditions, such as the preparation of *Bananenstrudel* (banana strudel) or *Reisknödel* (rice dumplings) for example, but also by acculturating Austrian-German food such as the *Wiener Schnitzel*[34] more recently.

### Sample

The fieldwork was conducted simultaneously by the first, second and third authors between July 2008 and January 2009 in Australia (A) – in the metropolitan areas of the federal states of New South Wales (Sydney), Queensland (Brisbane, Gold Coast), Victoria (Melbourne, Beechworth, Perisher Blue), Southern Australia (Adelaide) and the Northern Territory (Alice Springs) – in Brazil (B) in Treze Tílias, and in Peru (P) in Pozuzo. In order to collect comparable data, the same interview technique was used in accordance with field guidelines developed by the project members. In all research areas, informants – either first-generation Tyroleans or descendants of Tyroleans – were initially randomly picked and interviewed. After the interview, each informant was asked to recommend Tyroleans or people of Tyrolean descent who are familiar with the topic in question. Interview partners were then chosen from this pool of recommended people (snowball sampling).

The intention was to keep the sample evenly distributed among men and women. Overall, in all areas of research more women were interviewed than men (Table 2). The eligibility criteria for the sample included being at least 18 years old, of Tyrolean descent (first, second and up to fourth generation) and permanently resident in the area of migration. In all, for networks of food knowledge, 30 people (A: 13; B: 8; P: 9) aged from 20 to 87 (arithmetic mean: A: 53, SD = 16; B: 44, SD = 22; P: 57, SD = 15) were interviewed. As regards medicinal plant knowledge, 26 networks (A: 11, B: 7, P: 8) were compiled and the respondents’ ages ranged from 32 to 95 (arithmetic mean: A: 45, SD = 11; B: 67, SD = 21; P: 54, SD = 16).

**Table 2:** Sample of respondents for personal networks of food (n = 30) and medicinal plant (n = 26) knowledge in Australia, Brazil and Peru

**Domain**		**Food**								**Medicinal plants**							
**Areas of research**		**Australia**		**Brazil**		**Peru**		**Total**		**Australia**		**Brazil**		**Peru**		**Total**	
**Number/percentages**		n	%	n	%	n	%	n	%	n	%	n	%	n	%	n	%
**Sex**	**female**	8	62	5	63	5	56	18	60	5	46	3	43	4	50	12	46
	**male**	5	38	3	37	4	44	12	40	6	55	4	57	4	50	14	54
**First language**	**German**	13	100	7	88	8	89	28	93	11	100	7	100	4	50	22	85
	**other**	-	-	1	12	1	11	2	7	-	-	-	-	4	50	22	85
**School education**	**primary**	2	15	2	25	4	45	8	27	4	36	3	43	6	75	13	50
	**≤high school**	9	70	2	25	3	33	14	46	5	46	3	43	-	-	8	31
	**university**	2	15	4	50	2	22	8	27	2	18	1	14	2	25	5	19

**Figure 2:**
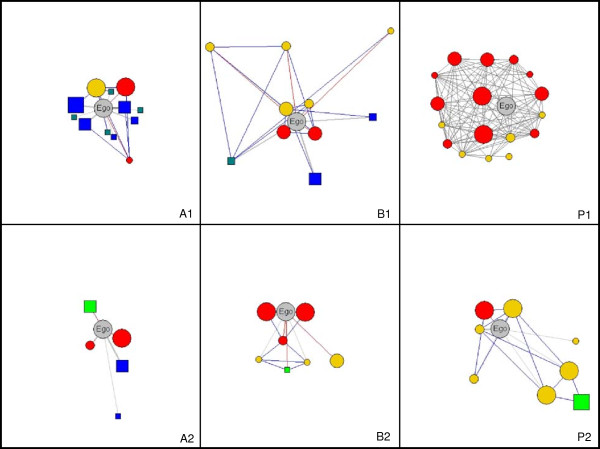
**Examples of digitalised personal network maps from Australia, Brazil and Peru.** Knowledge of food (A1: Australia, B1: Brazil, P1: Peru) and knowledge of medicinal plants (A2: Australia, B2: Brazil, P3: Peru). Colours of network cards: grey - ego; red - individual alters already deceased; yellow - individual alters still alive; dark green - print media; light green - institutions. Lines: red - no contact at present; blue - contact between alters; grey - contact between egos and alters. Name labels removed for anonymisation.

In Australia all informants were first or second-generation Tyrolean. Five of these interviewees about food knowledge were employed as chefs or ran their own restaurant at the time of the interview. All of them are first-generation migrants from Tyrol. One second-generation informant teaches cookery in a state school. All the other informants were recommended by members of the Austrian Club in Adelaide, Sydney, Gold Coast and Melbourne. None of these informants is specifically associated with the domain other than cooking on a regular basis at home. Figure 2 (A1) displays the network for food knowledge of a female informant who migrated to Australia in the 1950s aged 20 and married an Australian. She continued to prepare the Tyrolean-Austrian dishes she learned during her childhood in Tyrol from her mother and sister, whom she still contacts about Tyrolean recipes. After her marriage she learned from her mother-in-law. Nowadays her cooking is greatly influenced by the various cooking styles offered by Australian cuisine and she keeps her knowledge up to date by reading cookery magazines and watching TV cookery shows. In Australia, all informants concerning knowledge of medicinal plants are first-generation migrants from Austria. None of the interviewees could be considered to be an expert in the field. Figure 2 (A2) displays the network of medicinal plant knowledge of a male informant who migrated to Australia in the late 1960s as a chef while in his early twenties. After working for top hotels he started his own business producing culinary condiments. He learned a great deal about medicinal plants from his grandmother, his father and a nun teaching in his school. Nowadays he consults popular books about the European healing tradition to acquire knowledge of medicinal plants.

In Brazil the informants were mainly first, second and third-generation Tyrolean or other Austrians who emigrated with the group. In Treze Tílias, two interviewees about food knowledge work or used to work in restaurants and hotels. Two informants produce and sell typical Tyrolean food and drink, such as apple strudel and schnapps. These four interviewees were considered experts in Tyrolean food by Treze Tílians. The other four interviewees are not associated with restaurants or the sale of food products. Figure 2/B1 displays the network of food knowledge of a daughter of Tyrolean migrants who was born in the 1950s in Treze Tílias where she has lived all her life. She used to own a culinary establishment in Treze Tílias selling mainly typical Tyrolean dishes such as apple strudel, fruit bread and schnapps.

Among the seven interviewees about medicinal plants in Treze Tílias, none were experts on medicinal plant use except for using medicinal plants casually for domestic purposes. Figure 2/B2 displays the network of medicinal plant knowledge of a son of Tyrolean migrants born in the 1940s, who is now the owner of one of the biggest hotels in Treze Tílias. He is married to an Austrian woman and visits Austria often. He learned most of what he knows from his parents and an Austrian nurse who was among the first group of migrants to arrive in Treze Tílias. She used to teach people how to use medicinal plants since the local medical facility had limited medicinal supplies. Nowadays, if necessary, the informant consults a woman with a Brazilian background who is considered to be an expert in the field of medicinal plants. He also contacts another woman who used to work at the medical facility as well and people who still work there now.

In Pozuzo all informants have Austrian or German ancestors, are associated with Tyrolean descendants or are well integrated into the “Tyrolean” community of Pozuzo and have at least some knowledge of the German dialect “Tirolés”. The interviewees about food knowledge are affiliated to restaurants (1) or hotels (3), or were considered by local people to be experts on Tyrolean food (5). Figure 2/P1 displays the network of food knowledge of an elderly woman recommended by many informants. She used to help as a kitchen assistant preparing food for wedding receptions where she gained a great deal of knowledge of Tyrolean food. She mainly learned from people who were in the Tyrolean community. Most of the people she mentioned have died.

All Pozuzian informants about medicinal plant knowledge were considered by local people to be experts in medicinal plants. Five of these informants took part in medicinal plant projects in schools, research institutions or projects in a development context. One informant is explicitly considered to be a healer - a so-called *Cocoma –* who is married to a Tyrolean descendent. He gained his knowledge from his grandmother, a healer, and from using mind expanding drugs (*ayahuasca)*. Figure 2/P3 displays the network of the medicinal plant knowledge of a woman in her forties who obtained most of her knowledge from family members, from a person working on her family’s farm, from acquaintances who worked in plant-related projects and by attending a workshop on ecological tourism and medicinal plants organised by the EU-Peru programme for alternative development in the Pozuzo-Palcazú area (PRODAPP).

### Data collection

In advance of the interviews, all the informants received formal letters explaining the content of the project and assuring them that any data would be used confidentially. Oral informed consent was obtained from the informants for the collected data and any accompanying images to be published. In both domains (knowledge of food and medicinal plants) personal networks of knowledge sources were collected and visualised in order to discuss their characteristics and the context of knowledge transmission. Finally, socio-demographic data (name, address, sex, age, place of birth, mother tongue, year of migration, parentage, education) were recorded. Interview notes were written on prepared forms and all interviews were audio recorded (Olympus Digital Voice Recorder DS-30) with the respondents’ permission. Although the content of knowledge about food and medicinal plants is mentioned, this paper focuses primarily on the sources of knowledge of food and plants.

Simple white paper sheets were used to create network cards in order to collect quantitative data. Additionally qualitative interviews were conducted to allow informants to give further information related to the actors named in their network on knowledge sources. Qualitative hermeneutic analysis of the narratives and quantitative analysis of the ego-network cards were then combined to analyse data. Data for the content of knowledge of food and medicinal plants comes from quantitative data collected by free listing and semi-structured intreviews. Furthermore, participant observation on some particular dishes (Tyrolean dumplings and strudel) was accomplished. Still, the content of the article shows a clear emphasis on the interpretation of quantitative data by analysing personal network cards of food and medicinal plants.

All the information collected is self-reported and there is an awareness that the study does not provide a complete picture of the reality. However, it can be regarded as a pilot methodology for providing indications in future ethnobotanical research.

There was no obligation to obtain research permits for the research areas, as work was not being undertaken with indigenous communities, and voucher specimens were not collected. The Code of Ethics of the International Society of Ethnobiology (ISE) was followed.

#### ***Personal networks - name generator***

A personal network is a social network perceived from the perspective of the respondent (ego) and encompasses social ties to people or other types of actors (alters) [50]. It differs from the whole network (sociocentric) that features the pattern of relationships among all network members in a bounded population. Usually, personal networks (egocentric networks) focus on the social relationship to alters – most often people – with regard to a specific question. In this study, personal networks were collected based on the “Wellman approach” [51]. A *single name generator* was used to ask respondents (referred to throughout this paper as “egos”) to name sources of knowledge (referred to throughout this paper as “alters”) in the respective domain. Alters may include individuals (*e.g.* family, friends and acquaintances) and non-individual sources (*e.g.* television, the internet and cookery books). The single name generator for the domain of food was: “Where does your knowledge come from about the preparation of food, drinks and preserved food?” and for the domain of medicinal plants: “Where does your knowledge of medicinal plants come from?”. The interview partners (egos) were encouraged to name more alters using the name generator repeatedly and space was given for narratives to recall alters from different stages of the respondents’ life. The name generator was used on average five to six times per interview in all research areas. *Name interpreter* questions were inserted into the narrative in order to elicit additional information on age, sex, the role of the alters mentioned, relationship ties, contact frequency and ties between alters. The informants named all the sources from whom they had gained at least some knowledge of food and medicinal plants during their whole lifetime, including deceased ancestors for example (mothers/fathers *etc.*).

“Contact frequency” encompasses all the contacts the informants had mentioned during the interview. In this study only the contact frequency of the informants to their individual alters at the present time was of interest. The informants were therefore asked:” How often do you have contact with the person named at the moment?”

The roles (*e.g.* neighbour, friend, work colleague) were not pre-defined to avoid pre-categorisation and leave room for people’s own interpretation [52,53]. The name of alters plus additional information was written on a prepared paper form to provide basic information for the visualisation of the network cards.

#### ***Visualisation of personal networks on network maps***

**Figure 3:**
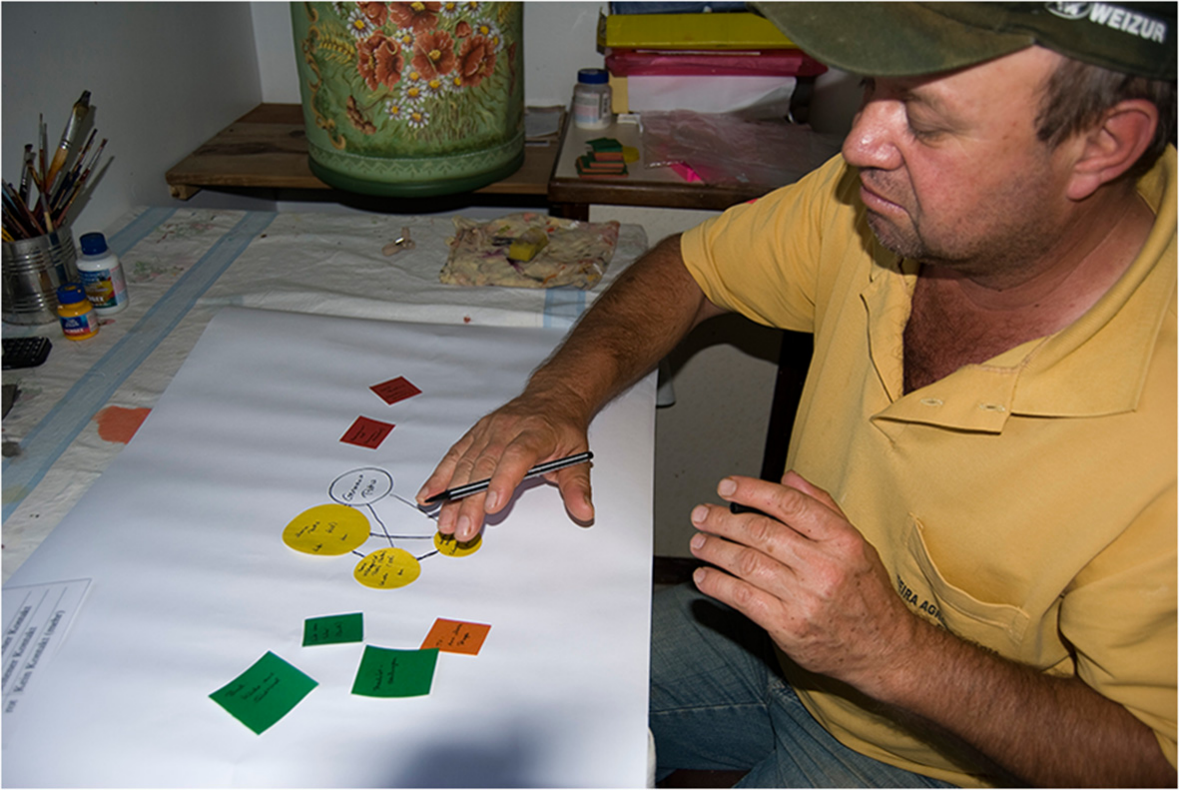
Brazilian respondent (ego) of Tyrolean origin creates a map of his personal network (alters) of food knowledge (Kuhn 2008).

When the informants were unable to recall any more alters, the personal network was visualised using A0-paper to create a network map (Figure 3). Pre-defined paper network cards (Table 3) were placed on the network map to record complex relationship structures and obtain a clear illustration of the interviewee’s network in order to support memory recall during the interview [54-58]. The pre-defined paper network cards varied in form and colour to represent different types of alters (Table 3). The four different sizes of paper cards indicated the amount of knowledge transmitted to the ego by each alter (Table 3).

**Table 3:** Elements used to visualise the personal networks on paper sheets

**Element**	**Visualisation on paper**
Ego (respondent)	Round yellow card in the centre of an A0 paper sheet
Alter (type of knowledge source)	Person: round yellow card
	Audiovisual: orange rectangular card
	Print media: brown rectangular card
	Institutions and organisations: red rectangular card
Amount of knowledge	Size of alters’ card in cm (Ø 4 = not much, Ø 5 = some, Ø 6 = much, Ø 7 = very much) to represent different amounts of knowledge
Relationship ties	Expressed through the distance from the ego card to all alters’ cards allocated by ego
Contact frequency (ego to alters)	Three lines (≡): often
	Two lines (=): occasionally
	One line (-): rarely
	One line (- red colour): no contact († dead)

Hence, the visualised personal network displays four different levels of information on the network structure: first the network of the different knowledge sources on the specific domain of the informants’ entire lifetime; second the closeness felt by the informants to the individual knowledge sources they had named; third the frequency of contact at the moment between the interviewees and the named sources of knowledge, and fourth the amount of knowledge transferred.

For the visualisation on the network map, a round piece of yellow card was placed in the centre of the A0 blank paper sheet to represent the respondent (ego). Then paper cards were chosen by the informant according to the type of named alter and the amount of knowledge transferred (Table 3). Additional information (name, age and social role) collected during the interview process was written on the cards. Then all the paper cards for human alters (referred to as “individual alters”) were arranged by the ego (=informant) around the ego paper card in the centre according to the emotional closeness felt by the ego for individual alters. The nearer the card was placed to the ego, the closer the relationship tie. To indicate the frequency of contact at present, lines were drawn between the ego and all the individual alters who are acquainted with one another. To display the intensity of the frequency of contact at present, respondents could choose one of four different possible indications (Table 3).

The frequency of contact at present refers to “current” existing networks, therefore dead alters were considered separately in the relations category of “no contact at present”.

**Figure 4:**
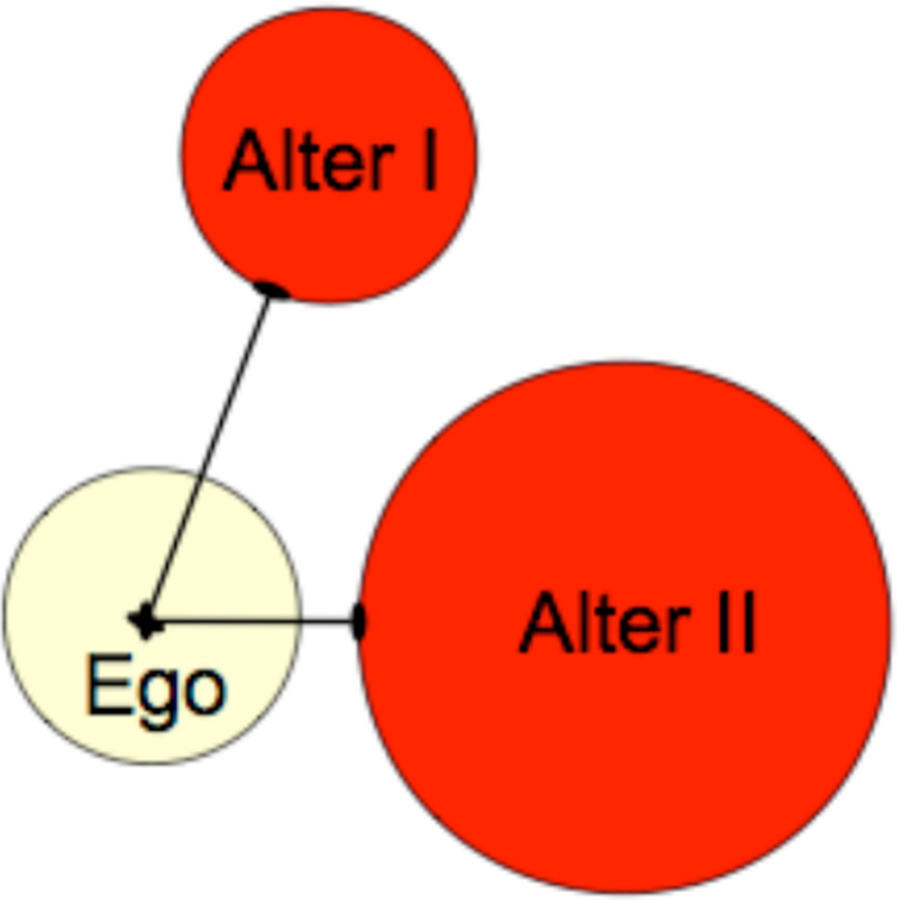
Demonstration of the measurement of the distance in between ego and alter.

The different card sizes were considered when calculating the distance parameter. Figure 4 demonstrates the calculation of the distance parameter where the distance from the ego to the central point of alter I and II is the same. Therefore, the distance parameter was measured (in cm) from the central point of the ego card to the edge of each alter card (Figure 4).

Other alters such as print media, audiovisual media, organisations or institutions (referred to as “non-individual alters”) represented by rectangular, coloured cards were placed around the ego card in the same manner. Once the network map had been completed, all the cards were glued onto the paper sheet and the whole network map photographed. EgoNetQF software [59] was used to digitalise the network maps in order to present the personal networks anonymously (Figure 2).

### Data analysis

The network maps serve as original data sheets. Alters include individual and non-individual sources. Individual alters are individual people or, very rarely, a group of people *e.g.* a family or group of people that informants did not know or did not remember well and were therefore generalised, such as a family they used to visit or a group of hunters with whom they would spend a few days (Pozuzo). Non-individuals alters are: i) print media, *e.g.* books, magazines and newspapers, ii) audiovisual media, *e.g.* television, the internet and radio, and iii) institutions and organisations, *e.g.* schools, restaurants, clubs or medical institutions. The information collected was analysed using MS Excel [60], Pajek [61] and SPSS 16 [62] to perform univariate (descriptive) and bivariate (testing of the relationship) statistical analysis of the networks. In both the domains of food and medicinal plants, calculations were undertaken to determine the degree of personal networks including all alters, as well as the degree [63] of the personal network including individual alters only (referred to as the “personal network of individuals”) and the density [63]. The degree (size) of a network is the number of alters to which the ego is connected in a network. The density of a network is the number of lines in a simple network expressed as a proportion of ties that exist in a network out of all the possible ties. It is the degree of interconnectedness of network members, where high density reflects a network in which many members know one another and low density reflects a network in which few of them know one another. The degree is calculated by the formula Density = M/N(N-1)/2, where M is the number of realised ties among network members and N is the network size. A density of 1 implies that each alter is connected to every other alter. A density of 0 implies that none of the alters know any of the others [63]. By using the index of qualitative variation (IQV) two different diversity parameters of the personal networks (the diversity of individual alters and non-individual alters named, as well as the diversity of sex among individual alters in each personal network) were calculated. The IQV is a measure of variation among the categories of a qualitative variable. It is calculated by the formula IQV = K(N^2^-∑f^2^)/N^2^(K-1), where K is the number of categories (in both diversity measures = 2), N is the number of cases (*e.g.* four men and five women), and f is the frequency [64]. The factor ranges from 0 to 1 with a higher index indicating greater heterogeneity.

For comparison across different research areas, cross-tabulation, chi^2-^tests, Mann–Whitney U-tests and analysis of variance (ANOVA) were used. Correlation analyses were performed between the age of the egos and the number of alters (individual and non-individual) named. A *t*-test was performed to establish the significance of the difference between the means of the number of alters (individual and non-individual) mentioned by female and male egos. All narrative audio material was transcribed and analysed by qualitative content analysis [65] using Atlas.ti software [66].

### Practical notes on collecting data through personal network maps

During the process of collecting data, different challenges of a practical and theoretical nature were faced. Older egos had problems with eyesight, memory and comprehension and needed more assistance and explanations from the interviewers. It was challenging for egos to remember people’s names and professions if the emotional tie was not that strong. Sometimes egos could not accurately remember the names of books, websites or television programmes.

Overall, the interview method was successful. The use of paper sheets for data collection left room for improvisation which would not have been possible had a computer program been used. Due to the limited space on the network sheets, larger networks became slightly confusing. The name generator and interpreter provided enough inspiration for continuing narratives started by the egos. The visualisation of the personal network was hands-on work for the egos, where their efforts resulted in a concrete output. This kept egos attentive until the end of the interview and made them happy by remembering people they had not thought about for a long time. The quantitative approach has the advantage of simple statistical specifications and analysis which would have been difficult to visualise through a qualitative approach alone. The methods worked well to give an overall picture of processes of knowledge transmission and to allow a further, in-depth quantitative and qualitative questioning and work approach.

## Results

### Knowledge sources of food knowledge

#### ***Personal networks of food knowledge***

**Figure 5:**
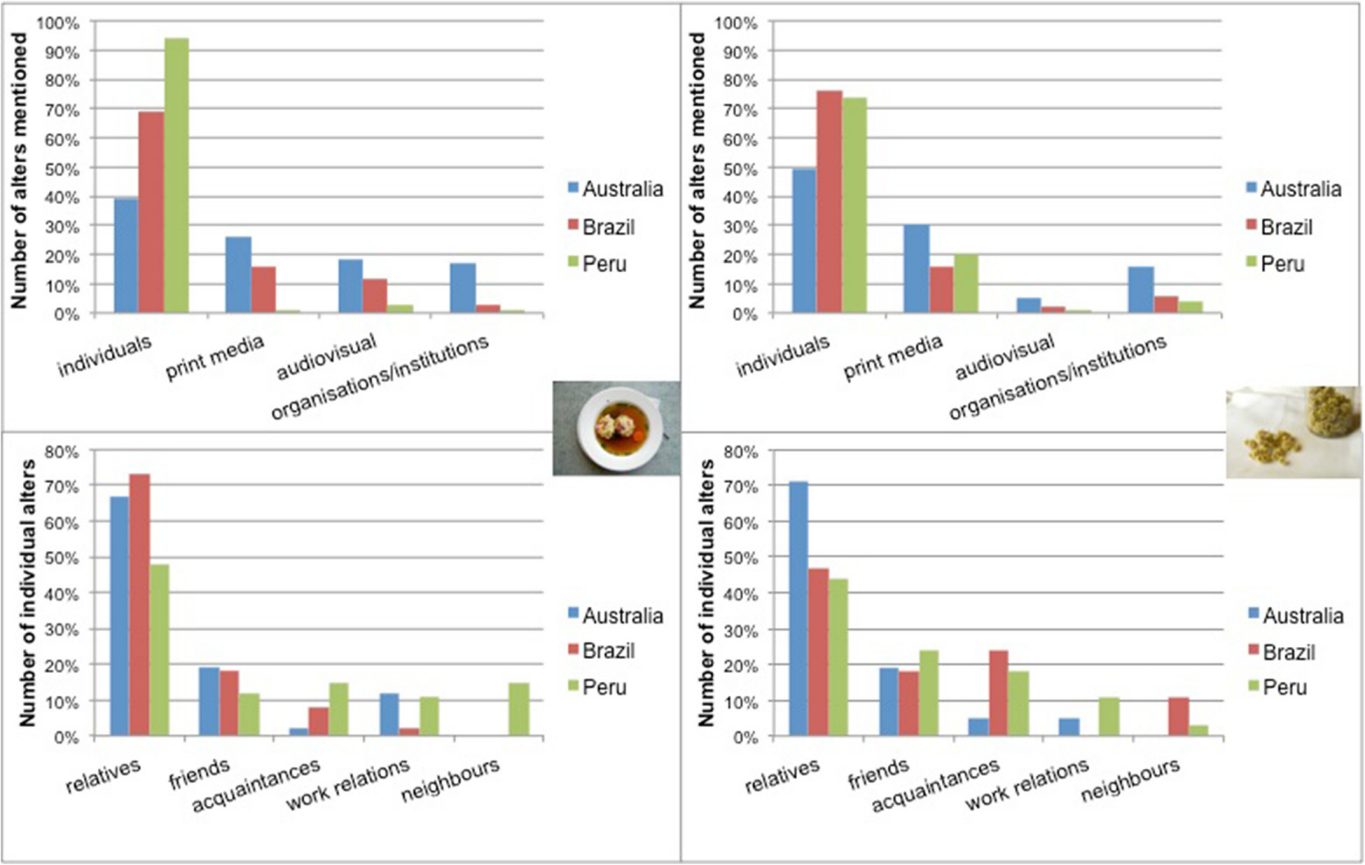
Top: alters (%) named in personal networks; bottom: type of individual alters only; left: networks of food knowledge (A: n = 13; B: n = 8; P: n = 9); right: networks of medicinal plant knowledge (A: n = 13; B: n = 8; P: n = 9).

In total, 335 alters (A: 107; B: 90; P: 138) were named by the egos (Table 4), with most alters named in Peru. The fewest alters (4) were cited in Australia. One respondent in Brazil recalled the maximum of 33 alters (Table 5). However, there is no significant difference in the size of the networks (total number of individual and non-individual alters mentioned by ego) between the areas of investigation (Table 6). The IQV calculated for the diversity of alters (frequency of individual compared to frequency of non-individual alters in each country) is highest in Australia (Med = 0.9, Stddev = 0.09) followed by Brazil (Med = 0.78, Stddev = 0.17). In Peru the IQV for the diversity of alters is lowest (Med = 0.23, Stddev = 0.29), which means that the networks there are more homogenous than in Australia and Brazil. There is no significant difference between Australia and Brazil in the IQV calculated for the diversity of alters, but there was a significant difference between Australia and Peru and Brazil and Peru (Table 6). Individual alters (91%) were mainly named in Peru in comparison with Australia (39%) and Brazil (69%) (Table 4, Figure 5). In Peru six of the networks consisted of individual alters alone, whereas in Australia and Brazil non-individual alters were always named in addition to individual alters. This indicates that in Peru, and to a lesser extent in Brazil, food knowledge transmission through individual alters is crucial. In Australia personal networks of individuals are small and the number of ties realised is low compared to Brazil and Peru where many possible ties are realised (Table 5). Due to the low number of individual alters, the density factor is of limited statistical significance. Networks are densest in Brazil (0.83) and individual alters mostly know one another. In Peru networks are not as close (0.53) and individual alters know one another to a lesser extent. Due to the low number of individual alters, mainly family members, the density in Australia is not very meaningful for statistical comparison. There is no correlation between the number of alters (individual and non-individual) cited and the age of egos (n = 30, p = 0.815, r_Pearson_ = 0.045). The *t*-test indicated that there is no significant difference between female and male egos in the number of alters (individual and non-individual) cited (n = 30, p = 0.262).

**Table 4:** Number (n = absolute number of informants interviewed, % = percentage of informants interviewed) of several variables of alters in personal networks of the two domains of food and medicinal plants in Australia, Brazil and Peru

**Domain**		**Food**								**Medicinal plants**							
**Areas of research**		**Australia**		**Brazil**		**Peru**		**Sum**		**Australia**		**Brazil**		**Peru**		**Sum**	
		**(n = 13)**		**(n = 8)**		**(n = 9)**		**(n = 30)**		**(n = 11)**		**(n = 7)**		**(n = 8)**		**(n = 26)**	
**Number/percentage (n/%)**		**n**	**%**	**n**	**%**	**n**	**%**	**n**	**%**	**n**	**%**	**n**	**%**	**n**	**%**	**n**	**%**
**Alter’s sex**	Male	13	31	8	13	44	35	67	29	4	19	13	46	35	56	52	43
	Female	29	69	54	87	82	65	167	71	17	81	25	89	28	44	70	57
**Alter’s age**	<20	-	-	1	2	-	-	1	-	-	-	-	-	1	2	1	1
	20-29	-	-	10	16	6	5	16	7	-	-	-	-	2	3	2	2
	30-49	8	19	12	19	23	18	43	18	3	14	1	3	11	17	15	12
	50-69	10	24	24	39	43	33	77	33	1	5	12	32	8	13	21	17
	= > 70	9	21	9	15	17	13	35	15	5	24	7	18	10	16	22	18
	dead	15	36	6	10	41	32	62	27	12	57	18	47	31	49	61	50
**Alter’s age in relation to ego’s**	younger than ego	3	11	11	20	28	32	43	24	-	-	4	20	6	19	10	16
	same age	11	41	19	34	39	44	69	39	5	56	8	40	14	44	27	44
	older than ego	13	48	26	46	22	25	66	37	4	44	8	40	12	38	24	39
**Role of alters**	relatives	28	67	45	73	63	48	136	58	15	71	18	47	29	44	62	50
	non-relatives	14	33	17	27	67	52	98	42	6	29	20	53	37	56	63	50
**Type of alter**	**individuals**	**42**	**39**	**62**	**69**	**126**	**91**	**234**	**70**	**21**	**49**	**38**	**76**	**63**	**71**	**122**	**67**
	household	-	-	-	-	4	3	-	-	-	-	-	-	3	3	3	2
	**print media**	**28**	**26**	**14**	**16**	**2**	**1**	**44**	**13**	**13**	**30**	**8**	**16**	**18**	**20**	**39**	**21**
	books	23	21	8	9	2	1	33	10	12	28	6	12	15	17	33	18
	journals	4	4	3	3	-	-	7	2	1	2	1	2	2	2	4	2
	magazines	1	1	3	3	-	-	4	1	-	-	1	2	1	1	2	1
	**audiovisual**	**19**	**18**	**11**	**12**	**4**	**3**	**34**	**10**	**2**	**5**	**1**	**2**	**1**	**1**	**4**	**2**
	internet	7	7	4	4	-	-	11	3	-	-	-	-	1	1	1	1
	TV	12	11	5	6	3	2	20	6	2	5	1	2	-	-	3	2
	radio/podcast	-	-	2	2	1	1	3	1	-	-	-	-	-	-	-	-
	**organisations/institutions**	**18**	**17**	**3**	**3**	**2**	**1**	**23**	**7**	**7**	**16**	**3**	**6**	**4**	**4**	**14**	**8**
	educational institutions	6	6	1	1	2	1	9	3	3	7	-	-	3	3	6	3
	hotels, restaurants	12	11	2	2	-	-	14	4	3	7	-	-	-	-	3	2
	ritual meeting	-	-	-	-	-	-	-	-	-	-	-	-	1	1	1	1
	**individuals**	-	-	-	-	-	-	-	-	**1**	**2**	**3**	**6**	-	-	**4**	**2**
	clubs																
	**Total**	**107**	**100**	**90**	**100**	**138**	**100**	**335**	**100**	**43**	**100**	**50**	**100**	**89**	**100**	**182**	**100**

**Table 5:** Size of personal networks and ties in food and medicinal plant knowledge in all areas of investigation

	**Country**	**Australia (n = 13)**				**Brazil (n = 8)**				**Peru (n = 9)**			
**Food**		**Med**	**Min**	**Max**	**SD**	**Med**	**Min**	**Max**	**SD**	**Med**	**Min**	**Max**	**SD**
	Size of personal network* (degree ego)	8	4	17	4	11	4	33	9	15	4	28	9
	Ties in personal networks	9	4	19	4	79	10	472	159	72	6	168	53
	Size of personal network of individual alters*	3	1	7	1	8	3	27	8	14	3	25	8
	Ties in personal networks of individuals	4	1	9	2	52	6	319	108	71	4	168	53
**Medicinal plants**	Size of personal network* (degree ego)	4	1	6	2	7	4	9	2	11	3	20	5
	Ties in personal networks	5	1	11	3	19	10	29	7	35	5	78	24
	Size of personal network of individual alters*	2	0	3	1	5	3	8	2	8	2	12	3
	Ties in personal networks of individuals	3	0	6	2	12	6	20	5	24	2	66	21

Coding: Med = median; Min = minimum; Max = maximum; SD = standard deviation; * = Ego excluded.

**Table 6:** **Statistical comparison (Mann–Whitney- *U *-Test) of the differences for the parameter mentioned in the first column for the two domains between the research areas (A), and for each domain separately between the research areas (B)**

	**A**			**B**					
**Statistical test**	Asymptotic significance (Mann–Whitney-*U*-Test)								
**Parameters**	**Food and medicinal plants**			**Food**			**Medicinal plants**		
**Research areas compared**	Australia and Brazil	Australia and Peru	Brazil and Peru	Australia and Brazil	Australia and Peru	Brazil and Peru	Australia and Brazil	Australia and Peru	Brazil and Peru
**Network size**	**0.045**	**0.001**	0.075	0.560	0.065	0.289	**0.008**	**0.002**	0.115
**Network size of individual alters**	**0.000**	**0.000**	**0.021**	**0.015**	**0.001**	0.147	**0.000**	**0.001**	0.092
**Network size of non-individual alters**	**0.008**	**0.000**	**0.039**	**0.025**	**0.000**	**0.002**	0.169	0.103	0.861
**Degree of female individual alters**	**0.025**	0.194	0.691	**0.012**	**0.002**	0.359	0.525	0.299	0.131
**Degree of male individual alters**	0.050	**0.001**	0.132	**0.042**	**0.000**	0.053	1.000	1.000	1.000
**Degree of kin individual alters**	0.977	0.081	0.135	0.818	**0.046**	0.053	0.749	0.532	0.816
**Degree of non-kin individual alters**	0.239	**0.000**	**0.049**	0.818	**0.031**	**0.033**	**0.049**	**0.004**	0.643
**IQV diversity of alters**	0.069	**0.000**	**0.030**	**0.065**	**0.000**	**0.002**	0.523	0.122	0.907
**IQV diversity of sex**	0.722	**0.010**	**0.025**	0.969	**0.040**	0.065	0.411	0.051	0.182

Bold type indicates significant differences between the research areas.

#### ***Individual alters in personal networks on food knowledge***

Informants recalled 234 individual alters (A: 42; B: 62; P: 126) as being transmitters of food knowledge (Table 4). The minimum of one alter was named by an informant in Australia and the maximum was cited by one person in Brazil (Table 5). The size of the personal networks of individual alters (total number of individual alters mentioned by ego) in Australia is significantly smaller than in Peru and in Brazil (Table 6, Figure 5). The proportion of female individuals as knowledge sources is higher than for males in all areas of investigation (Table 4). The IQV for the diversity of sex is relatively high in Peru (Med = 0.73, Stddev = 0.24) as networks of individual alters are more heterogeneous compared with Australia (Med = 0.37, Stddev = 0.43) and Brazil (Med = 0.34, Stddev = 0.4). Overall, female egos list women (A: 89%, B: 92%, P: 77%) more often than male egos list women (A: 33%; B: 63%; P: 41%). Male egos tend to name men in Australia (67%) and Peru (60%), but not in Brazil (36%) where male egos referred more often to female alters. There is a significant difference between the sex of the ego and the sex of the individual alters named for all areas of investigation (A: n = 46; p_chi-2_ = 0.000; B: n = 62; p_chi-2_ = 0.027; P:n = 126; p_chi-2_ = 0.000). These results indicate that knowledge about food tends to be transmitted between individuals of the same sex, especially among women.

Most individual alters identified in the three countries are between fifty and sixty-nine years old (Table 4). Egos in Australia and Brazil tend to name individual alters who are older than them (Table 4). Food knowledge in Peru is mainly transmitted between individuals of the same generation, as almost half of individual alters named are the same age as the ego (Table 4). However, in Australia and Peru the percentage of individual alters cited who were deceased (A: 36%, P: 32%) is high compared with Brazil, where people refer most to individuals of older generations who are still alive (Table 4).

Family relationships play a crucial role in the transfer of food knowledge (Figure 5). In all areas of research, alters from first-degree relationships (grandparents, parents, siblings, children) are very important in knowledge transmission, with parents being named most often (Table 7). In Peru half of the individual alters, and in Australia and Brazil almost three quarters, are family members, with parents being the most important knowledge transmitters (Figure 5, Table 7). As first-generation migrants in Australia are in less regular contact with their relatives in Austria, parent-in-law, brother-in-law and sister-in-law relationships are of more relevance in the transfer of knowledge about food practices compared to the other countries (Table 7). Friends are also frequently named in all areas of investigation (Figure 5, Table 7). In Peru, work relationships, informal local cooking get-togethers and cookery teachers are also important individual alters in the transmission of food knowledge (Figure 5, Table 7).

**Table 7:** Number (n = absolute number, % = percentage) of alters who are relatives and non-relatives in personal networks of food and medicinal plant knowledge in Australia, Brazil and Peru

**Domain**		**Food**								**Medicinal plants**							
**Research areas**		**Australia**		**Brazil**		**Peru**		**Sum**		**Australia**		**Brazil**		**Peru**		**Sum**	
		**(n = 13)**		**(n = 8)**		**(n = 9)**		**(n = 30)**		**(n = 11)**		**(n = 7)**		**(n = 8)**		**(n = 26)**	
**Number/percentage (n/%)**		**n**	**%**	**n**	**%**	**n**	**%**	**n**	**%**	**n**	**%**	**n**	**%**	**n**	**%**	**n**	**%**
**Relatives**		**28**	**67**	**45**	**73**	**63**	**48**	**136**	**58**	**15**	**71**	**18**	**47**	**29**	**44**	**62**	**50**
**1st degree**	Grandparents	5	12	4	6	8	6	17	7	3	14	-	-	5	8	8	6
	Parents/foster parents	11	26	12	19	15	12	38	16	7	33	10	26	11	17	28	22
	Siblings/stepbrothers and stepsisters	5	12	6	10	10	8	21	9	2	10	2	5	-	-	4	3
	Children	2	5	3	5	2	2	7	3	-	-	1	3	-	-	1	1
**2nd degree**	Aunts/uncles	-	-	6	10	13	10	19	8	3	14	1	3	2	3	6	5
	Cousins	-	-	5	8	1	1	6	3	-	-	-	-	1	2	1	1
	Nieces/nephews	-	-	-	-	1	1	1	-	-	-	-	-	-	-	-	-
	Parents-in-law	2	5	1	2	2	2	5	2	-	-	-	-	4	6	4	3
	Spouses/brothers or sisters-in-law	3	7	3	5	7	5	13	6	-	-	3	8	1	2	4	3
	Children-in-law	-	-	2	3	1	1	3	1	-	-	-	-	-	-	-	-
Distant relatives		-	-	3	5	3	2	6	3	-	-	1	3	5	8	6	5
**Non-relatives**		**14**	**33**	**17**	**27**	**67**	**52**	**98**	**42**	**6**	**29**	**20**	**53**	**37**	**56**	**63**	**50**
Friends		8	19	11	18	15	1	34	15	4	19	7	18	16	24	27	22
Acquaintances		1	2	5	8	19	15	25	11	1	5	9	24	12	18	22	18
Work relationships		5	12	1	2	14	11	20	9	1	5	0	0	7	11	8	6
Neighbours		-	-	-	-	19	15	19	8	-	-	4	11	2	3	6	5
**Total**		**42**	**100**	**62**	**100**	**130**	**100**	**234**	**100**	**21**	**100**	**38**	**100**	**66**	**100**	**125**	**100**

#### ***Non-individual alters in personal networks on food knowledge***

In Australia the percentage of non-individual alters cited as compared to individual alters cited was higher than in Brazil and Peru (Table 4) and there are significant differences in the size of networks of non-individual alters cited between all areas of investigation (Table 6). In Australia non-individual sources are most often cited by egos as sources of knowledge. Among non-individual sources of knowledge, print media are the most popular, followed by audiovisual media such as cookery shows on television, as well as the search for recipes on specific websites (Figure 5, Table 4). Five of the respondents interviewed in Australia were professional chefs and therefore the number of named alters, such as hotels and restaurants where food knowledge was acquired during apprenticeships, is high (Table 4). In Brazil books, television and internet use are frequently named as non-individual alters of knowledge transmission (Table 4), but there is still a significant difference between Australia and Brazil (Table 6) in the number of non-individual alters cited.

In Peru non-individual alters such as print and audiovisual media, organisations and institutions are significantly less important than in Australia and Brazil (Table 6).

### Personal networks of medicinal plant knowledge

For this domain, 182 alters (A: 43; B: 50; P: 89) in all were counted (Table 4). The smallest network was in Australia with only one alter named, while a maximum of twenty alters were named in Peru (Table 5). There is a significant difference in the size of networks (number of individual and non-individual alters cited) between Australia and Brazil and Australia and Peru, but not between Brazil and Peru (Table 6).

The IQV for the diversity alters is similar in all areas of investigation (A = 0.61, Stddev = 0.48; B = 0.58, Stddev = 0.36; P = 0.61, Stddev = 0.41) as individual alters are the main source for acquiring knowledge. Therefore no significant difference can be reported in the IQV calculated for the diversity of alters found between the research areas (Table 6).

In Australia the number of ties realised is low compared with Brazil and Peru (Table 5). Due to the low number of networks and the relatively low number of individual alters named, a statistical correlation of density is not meaningful. However, in Australia density in networks varies strongly due to the low number of individual alters named (p = 0.88, stddev = 0.48). Personal networks in Brazil (M = 0.8, stddev: 0.16) are denser than in Peru (0.66, stddev: 0.25) as individual alters named relate to one another more often than they do in Peru.

There is no correlation between the number of alters cited and the age of the egos (n = 26, p = 0.209, r_Pearson_ = 0.255). The *t*-test showed that there is no significant difference between female and male egos in the number of alters cited (n = 26, p = 0.094).

#### ***Individual alters in personal networks of medicinal plant knowledge***

Informants named 122 (A: 21; B: 38; P: 63) individual alters (Table 4). The smallest network was complied in Australia and the largest in Peru (Table 5). However, the size of personal networks of individual alters differs significantly between Australia and the other research areas, whereas there is no significant difference between Brazil and Peru (Table 6).

The IQV for the diversity of sex is low in Australia (Med = 0.34, stddev = 0.48) compared with Brazil (Med = 0.94, stddev = 0.40) and Peru (Med = 0.79, stddev = 0.21). Still, due to the high variance in networks, no meaningful statistical conclusion on the diversity of sex can be drawn. In Australia and Brazil, egos of both sexes mainly refer to female alters, female egos mainly refer to women (A: 92%; B: 79%; P: 46%) and male egos also tend to consult women more (A: 60%, B: 52%; P: 43%) for specific knowledge about medicinal plants. This does not account for people in Peru where there does not appear to be a preference for a specific sex of alters among respondents (Table 4).

There is no significant difference between the sex of the ego and the sex of the individual alters named in all areas of investigation (A: n = 23; p_chi-2_ = 0.089; B: n = 38; p_chi-2_ = 0.085; P: n = 23; p_chi-2_ = 0.511). These results indicate that knowledge of medicinal plants tends not to be transferred through a preferable sex in all areas of investigation.

Egos in all areas mainly refer to alters of the same age or older (Table 4). In all countries, almost half the individual alters named have already passed away (Table 4). More than 40% of the sources of knowledge named are relatives, with friends also named fairly frequently (Table 7). In all areas the parental generation appears to be most important in the process of knowledge transmission (Table 7). In Australia egos mainly refer to relatives and friends as knowledge sources. Furthermore, friendships and acquaintances, especially in Brazil and Peru, are a meaningful source of knowledge (Figure 5, Table 7).

#### ***Non-individual alters in personal networks of medicinal plant knowledge***

As with networks of food knowledge, egos in Australia tend to refer to non-individual alters more than individual alters compared with Brazil and Peru (Table 4). There is no significant difference between research areas regarding the degree of non-individual alters named (Table 6). In all areas of investigation, specific books on medicinal plants are popular alters named (Table 4). In Australia and Brazil egos also referred to television as knowledge sources. Two egos in Australia also referred to their schooldays in convent schools in Austria where the nuns who were teaching passed on knowledge about medicinal plants on country walks. In Brazil some people referred to healthcare facilities as places where knowledge of medicinal plants has been acquired. However, in all areas of investigation, media such as the internet and television are less important in the transmission of knowledge of medicinal plants compared with the domain of food.

### Amount of knowledge, frequency of contact at present and distance between egos and individual alters in personal networks of knowledge of food and medicinal plants

#### ***Personal networks of food knowledge***

**Figure 6:**
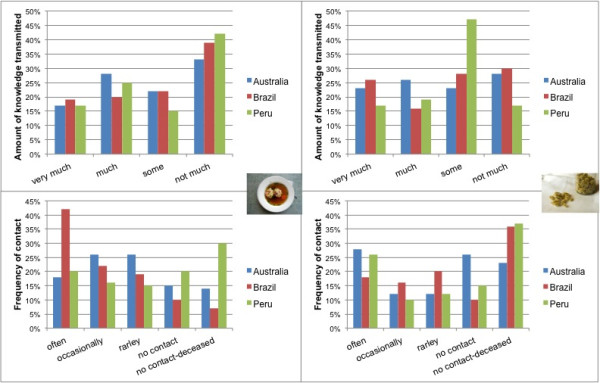
Top: amount of knowledge transmitted (%) in personal networks; bottom: frequency of contact (%); left: networks on food knowledge (A: n = 13; B: n = 8; P: n = 9); right: networks on medicinal plant knowledge (A: n = 11; B: n = 7; P: n = 8).

**Figure 7:**
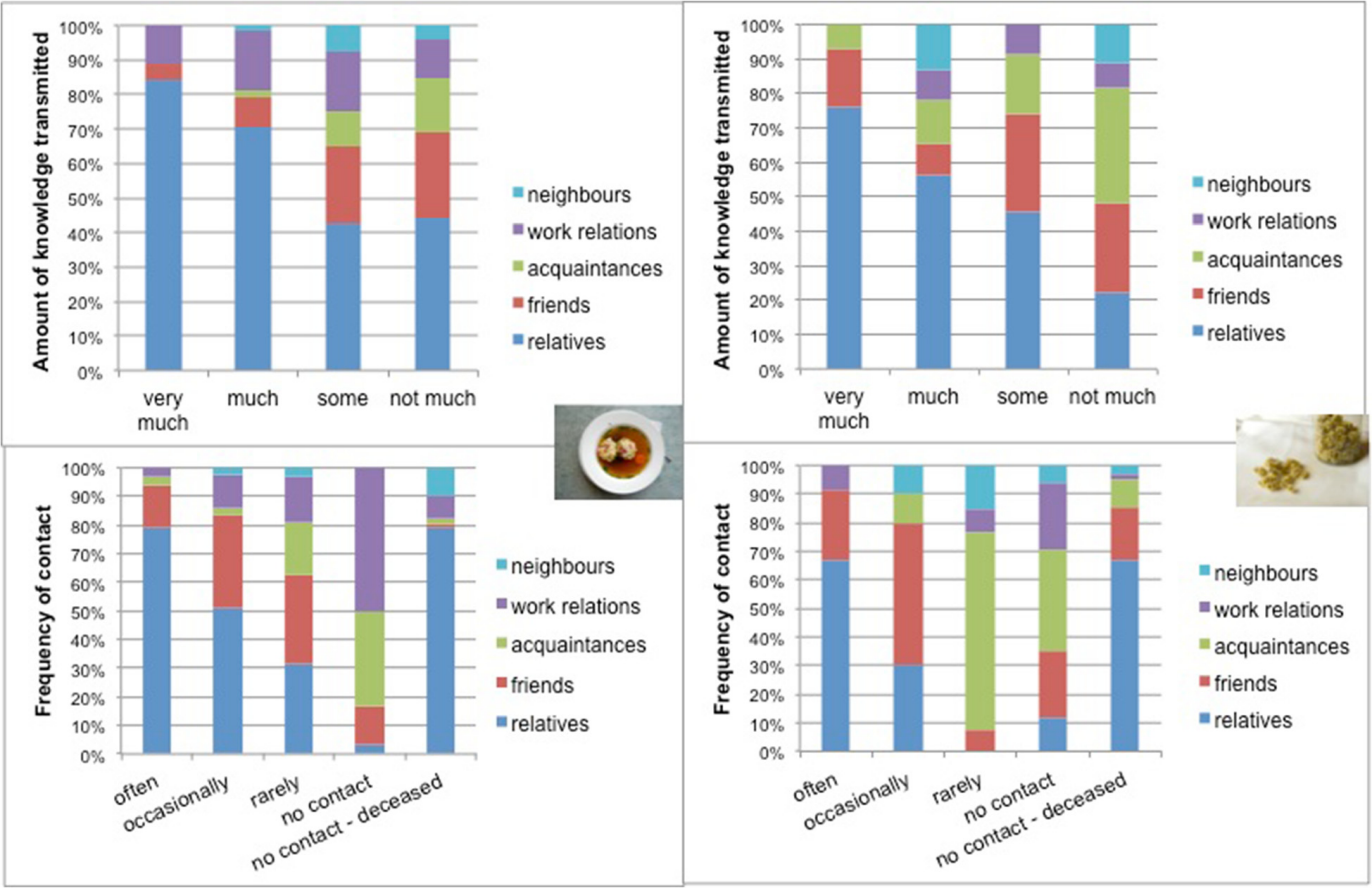
Top: amount of knowledge (%) transmitted in personal networks from individual alter roles to ego; bottom: frequency of contact (%) of ego with individual alter roles; left: networks of food knowledge (A: n = 13; B: n = 8; P: n = 9); right: networks of medicinal plant knowledge (A: n = 11; B: n = 7; P: n = 8).

Egos in all countries most frequently chose the smallest network card for alters (representing “not much” knowledge transferred) (A: 33%; B: 39% P: 42%), followed by the card “much” in Australia (28%) and Peru (25%) and “some” in Brazil (22%) (Figure 6). A trend can be seen that most alters provided “not much” knowledge, with a few alters passing on considerable amounts knowledge (Figure 6). “Very much” and “much” food knowledge was passed on through relatives, whereas “some” or “not much” knowledge was mainly transmitted through friends and acquaintances (Figure 7).

In Australia egos tended to contact alters either “occasionally” (26%) or “rarely” (26%), whereas in Brazil alters were “often” contacted (42%) by egos. The percentage of alters who were not in contact with one another was lowest in Brazil (10%).

Egos not only named individual alters from whom they gained knowledge now, but also during their childhood. Therefore some of the individual alters named had already passed away. Personal networks in Treze Tílias seemed to be built around existing contacts (“current networks”) and did not refer so much to the past (“remembrance networks”), as the number of alters named who have already died is lowest in Brazil (Table 4).

In Peru the percentage of no-contact relationships (20%) was higher than in other areas of investigation (A: 15%; B: 10%), which could be due to the fact that in Peru, in addition to many relatives, respondents talked about a number of distant relationships, such as occasional visitors and tourists (Table 7). Furthermore, the percentage of no-contact relationships due to alters who have already passed away (A: 14%; B: 7%; P: 30%) was highest in Peru, most probably because more egos over seventy were interviewed there as compared to Australia and Brazil (Table 4). However it could also indicate that knowledge of food is greatly related to Tyrolean heritage passed on by previous generations. In all, networks of food knowledge consisted of 66% active contact relations, 15% no contact relations and 19% of the individual alters mentioned already having passed away (Figure 7).

Correlations between the amount of knowledge and the frequency of contact at present indicate a tendency of egos to have contact “often” with alters who pass on “very much” and “some” knowledge (n = 334, p < 0.001) r^spearman^ = 0,287). If people who have already died are considered as missing values, this correlation coefficient increases even more (n = 272, p < 0.001, r^spearman^ = 0.312).

Statistical testing (ANOVA, post hoc) showed that egos tended to place individual alters in Peru further away (A, B and P: p = 0.000) as compared to Australia and Brazil (p = 0.918), which could be due to the fact that in Peru, in addition to many relatives, respondents also mentioned a number of distant relationships, such as occasional visitors and tourists (Table 7). Furthermore, variance analyses showed a tendency for placing individual alter cards representing relatives and friendships (p = 0.102) closer to the ego card as compared to the categories of acquaintances, work relations and neighbourhood (p = 0.000). According to post hoc Duncan testing by trend, two groups [67] can be distinguished: (i) “strong tie” relationships represented by relatives and friends (p = 0.102) and (ii) “weak tie” relationships including acquaintances and work relations (p = 0.825) and neighbours (p = 1.000).

In all areas of investigation “strong tie” relationships (relatives and friends) were contacted “often” or “occasionally“, whereas “weak tie” [67] relationships (acquaintances, neighbours and work relations) tend to be contacted “rarely” or there is “no contact at present” at all (Figure 7). This implies that most knowledge was transferred from alters who can be perceived as “strong tie” relationships with whom egos are in frequent contact, but also by alters who have already passed away (Figure 7).

Alters who had passed on larger amounts of knowledge (larger cards) tend to be placed closer to the ego, whereas alters who transferred less knowledge (smaller cards) are placed further away (n = 334, p < 0.001, r^spearman^ = 0.383).

#### ***Personal networks of medicinal plant knowledge***

In Australia and Brazil the smallest alter card indicating “not much” knowledge was the one most often chosen by egos (A: 28%, B: 30%) and in Peru the card expressing “some” knowledge (47%) was chosen most often (Figure 6). There is a tendency in all research areas for individual alters to pass on “not much” or “some” knowledge (Figure 6).

As with food networks, “very much” and “much” knowledge was mainly passed on through relatives, whereas “some” and “not much” knowledge tended to be transmitted by friends and acquaintances (Figure 7).

Egos not only named individual alters from whom they gained knowledge at the moment, but also during their childhood. Therefore some of these individual alters named had already passed away. In all networks the number of individual alters who had already passed away was high compared with the domain of food (A: 23%; B: 36%, P: 37%) (Figure 6). In Brazil and Peru they represent the largest group of individual alters, indicating that the networks of medicinal plant knowledge are more likely to be “remembrance networks”, especially in Brazil, than in networks of food knowledge. In Australia egos tended to contact alters either “often” (28%) or no longer had contact with their alters (26%), whereas in Brazil alters were contacted by egos at similar degrees in the “frequently”, “occasionally” and “rarely” categories or had “no contact” (Figure 6). In Peru, alters tended to be contacted “often” as compared to other categories (Figure 6).

There was a tendency (n = 121; p = 0.022; r_spearman_ = 0.208) for egos to have contact “often” with alters who passed on “very much” and “some” knowledge, and had contact “occasionally” and “rarely” with alters who passed on little knowledge.

Statistical testing (ANOVA, post hoc) showed that egos tended to place individual alters in Peru further away (A, B and P: p = 0.001) as compared to Australia and Brazil (p = 0.967), which could be due to the fact that in Peru, as in networks of food knowledge, in addition to relatives a large number of distant relationships were mentioned by the egos.

Variance analyses showed a significant difference between the relatives and acquaintances categories (p = 0.000) and the relatives and work relations (p = 0.017) in the distance at which the alter cards were placed to the ego cards. According to the post hoc Duncan analysis by trend (p = 0.082) two groups of relationships can be formed: (i) relatives and friends (“strong tie” relationships) and (ii) friends, neighbours, acquaintances and work relations (“weak tie” relationships). As with food, relatives are important in knowledge transmission, with friends and acquaintances being less important knowledge transmitters. In all areas of investigation, relatives tended to be contacted “often” and friends mostly “occasionally”, whereas acquaintances and neighbours tended to be contacted “rarely” (Figure 7).

There was a tendency to place the cards of individual alters who had passed on a greater amount of knowledge closer to the ego, whereas smaller cards were placed further away (n = 125, p = 0.004, r_spearman_ = 0.254).

### Summary of the comparison of both domains in all areas of investigation

**Figure 8:**
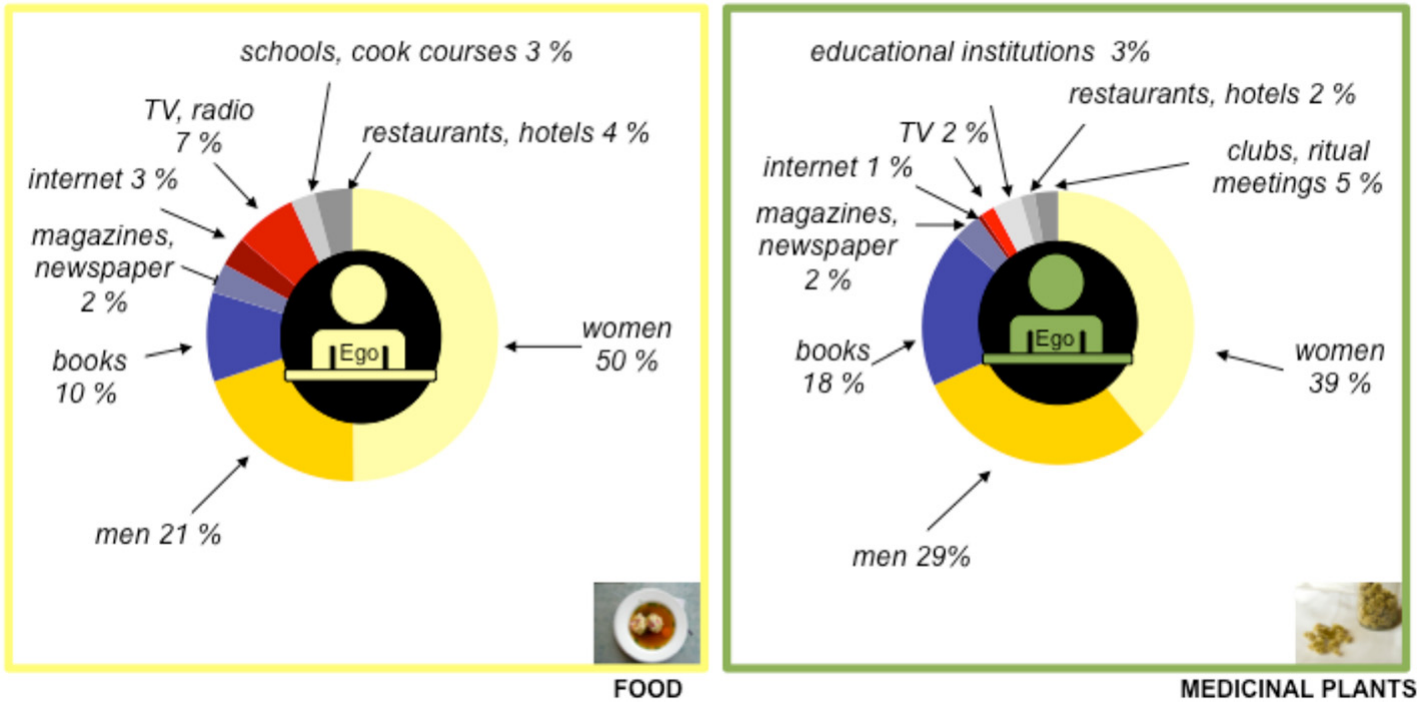
Different kind of alters (%) named in personal networks in all areas of investigation; left: networks of food knowledge (n = 30); right: networks of medicinal plant knowledge (n = 26); yellow tones: individuals; blue tones: print media; red tones: audiovisual media; grey tones: restaurants/institutions/organisations.

**Figure 9:**
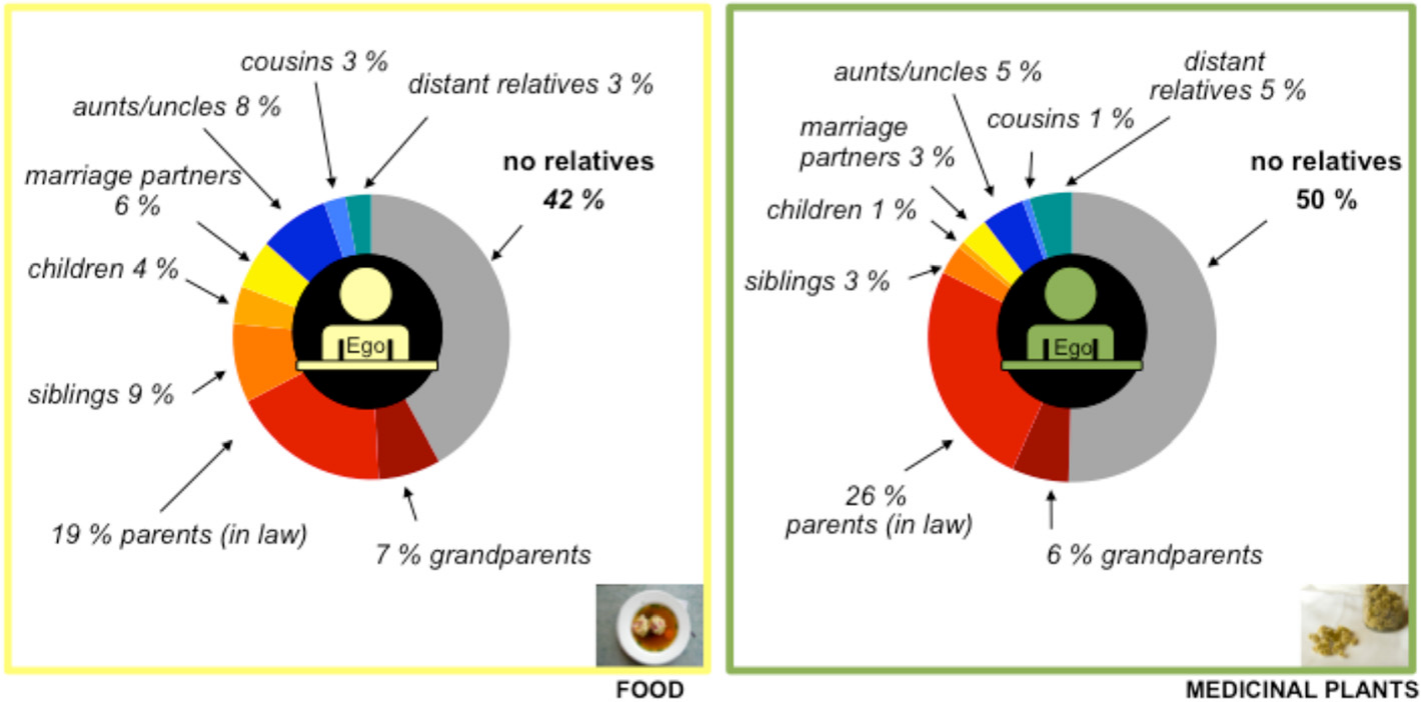
Types of relationships of all individual alters (only family relationships specified) (%) named in personal networks in all areas of investigation; left: networks of food knowledge (n = 30); right: networks of medicinal plant knowledge (n = 26); grey = no relatives, red tones = first-degree relative, blue tones = second-degree relative, green = distant relative.

Networks of food knowledge are significantly larger in size than networks of medicinal plant knowledge (p_Mann-Whitney-*U*-Test_ = 0.027). The percentage of non-individual alters is similar in both domains (p_Mann-Whitney-*U*-Test_ = 0.799). Also, comparing the percentage of individual alters, there is no significant difference (p_Mann-Whitney-*U*-Test_ = 0.149) between the domains. In both network domains individual alters represent the main source of knowledge (Figure 8). Relatives play a major role as alters in both domains (Figure 9, Table 7). The diversity of sex (IQV) is significantly different between the two domains (p_Mann-Whitney-*U*-Test_ < 0.001). Significantly more female alters were mentioned in networks of food knowledge than in networks of medicinal plant knowledge (p = 0.045). This is not the case for male alters (p_Mann-Whitney-*U*-Test_ = 0.879) (Figure 8). These results appear to indicate in particular that knowledge of food is mainly held and transferred by women. The quantity of knowledge transferred in networks of food knowledge is significantly greater than in networks of medicinal plant knowledge (p_Mann-Whitney-*U*-Test_ < 0.001).

### Reasons for and ways of acquiring knowledge

Qualitative content analysis provided insight into peoples’ motivations and ways of acquiring knowledge of food and medicinal plants. Informants’ statements in all areas of investigation indicate that knowledge transmission in the course of socialisation played an important role.

The knowledge gained during socialisation forms the knowledge base and contains basic techniques, such as heating the oven or skills to process the most important local resources, such as manioc and rice in Peru for example. These basics are further broadened through practice, questioning, observations and conservations which can be oriented towards acquiring knowledge about the preparation of a certain dish or incidentally by observing someone preparing food or hearing conversations about cooking and preparation techniques. Once the knowledge has been acquired, it is theoretically available, but the process of learning also needs a practical application. Food knowledge may be changed by the ego to match his or her needs and tastes. In this context informants talk about the innovations and experiments they undertake in their kitchen. Tyrolean chefs in Australia reported that traditional Tyrolean food became part of their professional cooking repertoire since the dishes seemed “exotic” on their menus and attracted people. Chefs pass on knowledge about how to prepare them to apprentices who do not have a Tyrolean background.

One reason frequently given by the egos for acquiring or rejecting knowledge in both areas was personal interest. Personal interest in food practices means that people are motivated to try out recipes and enjoy the results. Knowledge about the preparation of certain recipes is rejected out of a dislike for the recipes or cooking in general. Egos who are not interested in the specific domain and do not need it for their profession generally tend to have smaller networks. In these cases egos mainly learned from their parents or foster parents during childhood as they helped prepare dishes or became responsible for certain tasks related to cooking.

Personal interest in medicinal plants means that people are interested in plants and their healing qualities. People gain knowledge about medicinal plants to heal themselves or their relatives or due to a lack of professional medical assistance. If access to medical care is provided or there is no need to learn about medicinal plants because their health is good, the motivation to learn is low.

## Discussion

### Transmission of knowledge of food

This data shows that knowledge transmission concerning food through individual alters is crucial in Brazil and especially so in Peru, where a high proportion of the networks collected consisted of individual alters only. In all countries, alters from first-degree relationships (grandparents, parents, siblings, children) are very important in knowledge transmission, with parents being named most frequently. Therefore, socialisation processes are of utmost importance for gaining knowledge about food, and findings in this study support previous findings [2,3,7,12,14,16] that relatives play a crucial role in transmitting knowledge about food through the path of vertical knowledge transmission. In subsistence societies in particular, children learn most of their knowledge about their natural environment, notably ethnobotanical knowledge, during childhood [25].

Personal networks in Peru and Brazil tended to be bigger in size than in Australia, where networks tended to be more diverse, and non-individual alters such as print media, TV and the internet were named by all egos. Tyrolean migrants in Australia who mainly live in an urban context do not entertain networks of individuals to exchange knowledge about food on a regular basis. Living in urban, industrialised areas means they are in an environment with a very good infrastructure and access to media. Hence, it is suggested that this setting has led to a decrease in reliance on personal networks and favoured the consultation of non-individual alters such as media (TV, written word, internet)*.* This path of knowledge transmission can be perceived as a type of oblique transmission (one to many) [29]. Books, as Barth [9] states, lay out knowledge “as if it was context-free – a mode that collapses historical time in acquiring knowledge, elaborates taxonomies and prizes coherence”. Printed books also allow a more detailed and accurate transmission of knowledge in a persuasive way [27,68]. The consultation of books requires sufficient knowledge of language, terms, concepts and basic cooking skills [69]. Books are addressed to a certain group of people or social class and enable intercultural exchange between both ethnical groups and societal classes [68]. However, the type of oblique transmission through the written word from one to many may favour rapid cultural change within a group [27,28].

In Australia and Brazil, most knowledge about food is passed on by older relatives (parents, grandparents and siblings). Therefore the most important mechanism in knowledge transmission is vertical (parent–child). In Brazil, besides friendships and acquaintances, attending cookery courses and audiovisual media are relevant knowledge sources. Although informants did not mention culinary establishments explicitly as knowledge sources, they play an important role in the tradition of food knowledge as they communicate the concept of “Tyrolean food” to a broader public [70]. Therefore, knowledge about food is transmitted through dishes in restaurants, hotels and during celebrations they have organised. Informants in Treze Tílias have a preference for exchanging knowledge with people they feel close to. Unlike in Australia and Treze Tílias, in Pozuzo the path of horizontal knowledge transmission is relatively well developed in addition to vertical transmission. In Pozuzo individuals named within networks know each other to a lesser extent than in Brazil since it is not just community members who are knowledge sources, but individuals not permanently living in Pozuzo as well, such as teachers, visitors and tourists. Therefore in Pozuzo the low density of networks reflects the influence of regular short-term visitors from Tyrol who contribute new knowledge about food.

In Peru, as in Brazil, horizontal transmission becomes more significant during the informants’ adolescence as they turn more frequently to non-parental sources of knowledge, such as peer interactions or consultation of media [71]. Since Pozuzo remained geographically quite isolated for a long time due to the lack of transport and is still not always easy to reach, this remoteness might have favoured the reliance up to now on personal relations in gaining knowledge. Nowadays in Pozuzo, the tendency to exchange knowledge through new communication facilities (TV, radio, internet) is increasing due to higher educational standards and improved technical infrastructure [34], especially among people in the younger generation. In this study, the informants in Peru were mainly over fifty-five years old. However, in future research it will be vital to have a wider sample that covers all ages in order to trace sufficiently how society as a whole is changing. In Peru books were seldom named as sources of food knowledge. The dominant oral mode of knowledge transmission is a highly dynamic process and there are many very flexible and intracultural variations with innovations likely to happen, as no written documents exist as a reference. Transformations in knowledge occur through neglect, adaptation, improvement and new creations. Appadurai [68] observed that a merely oral transmission results in a specific regional cuisine, as is the case in Pozuzian cuisine [34] which is highly flexible, dynamic and characterised by innovations. Taking the example of “Tyrolean dumplings”, it was observed that dumplings in Australia and Brazil are prepared in the “Tyrolean way”. In Peru the bread-based dumplings changed to rice-based dumplings or the traditional filling of the “*strudel*” changed from apples to bananas. Both these changes can be seen as such a type of innovation due to adaptation to the new surroundings.

The proportion of females transmitting knowledge is higher than males in all areas of investigation. Therefore, as stated in previous research [69,72,73] on food knowledge, women play a crucial role in transmitting knowledge. These findings indicate that food knowledge tends to be transferred between individuals of the same sex.

### Transmission of knowledge of medicinal plants

Networks of food knowledge are geared towards existing networks which are currently active compared with networks of medicinal plant knowledge where people make numerous references to alters who have died and who are most often close relatives.

As in networks on food knowledge, in Brazil and Peru alters in networks of medicinal plant knowledge are mainly individuals. The role of relatives as knowledge transmitters is more pronounced in Australia than in Brazil and Peru, where egos also referred to friends, acquaintances, work relationships and neighbours as knowledge sources.

In all areas of research, egos refer to specific books as knowledge sources, especially in Australia. These findings may indicate that in future medical choices will progressively be influenced by written documents and media not only in industrialised places but also in the world’s remotest areas [27]. In Brazil and Peru, relatives as knowledge sources are less important than in food networks, and the proportion of alters that are the same age as egos is more or less equal. In line with most of the studies carried out on ethnobotanical knowledge [2,8,24,26,27,29], it is suggested that vertical and horizontal paths of knowledge transmission are both pronounced in this domain. Vertical transmission tends to be more dominant earlier in life, and knowledge gained later in adolescent life is mostly diffused through the horizontal path by consulting peers or experts (healers) in the field. Networks in Australia are significantly smaller than in other research areas. These results correspond to the findings of a comparative study [22] on the transformation of medicinal plants among Tyrolean migrants. According to these results, Tyrolean migrants in Australia mainly refer to knowledge they gained in vertical transmission processes during childhood. Tyrolean migrants in Australia – unlike Tyrolean migrants in Brazil and Peru – had a similar knowledge of medicinal plants as Tyroleans in Austria did. Consensus on the knowledge of medicinal plant taxa was higher between Tyrol and Australia than between Tyrol and Brazil and Peru, but the number of medicinal plants they listed during freelisting was much smaller as not much knowledge had been added to their basic knowledge since their departure [22]. However, data suggests that vertical transmission is dominant for highly shared knowledge, and new knowledge is mainly added through horizontal and oblique paths [25]. Overall, Tyrolean migrants and their descendants in Brazil and Peru have greater knowledge of medicinal plants than Tyrolean migrants in Australia. Migrants in Brazil and Peru had learned more about medicinal plants in their country of arrival by incorporating new plants into their traditional knowledge of medicinal plants by adopting and substituting medicinal plants to medicate diseases [22].

As proved in other studies on medicinal plant knowledge [8,24,30], knowledge of medicinal plants is mainly transmitted through women. In Peru the portion of male knowledge transmitters is slightly higher than in Australia and Peru. A correlation between age and the knowledge and use of medicinal plants has been proven in a survey conducted in Oaxaca, Mexico by Giovannini *et al.*[74].

### Food versus medicinal plant knowledge

A comparison of the two domains remains difficult as there are several issues that might affect why the two domains seem to follow different patterns. Knowledge about food is universal – a day-to-day activity – as everyone needs to eat on a regular basis, although it depends upon many factors (such as age and sex) as to how pronounced this knowledge is. Food is essential and therefore everyone has knowledge of food to a varying extent. The knowledge of food among Tyrolean migrants is influenced by the process of migration, the new social networks established, the strength of ties maintained with the country of origin and the availability of certain ingredients. Furthermore, the degree of exposure to the host culture may have influenced taste preferences and changes in consumption patterns and food preparation for example. Transmission processes are part of the collective memory of a group which is formed by ethnic and regional attributes and the power structure of a group in society [34].

The tradition of medicinal plant knowledge is not only challenged by the processes that are evoked by the migrational event, but also by increasing industrialisation where the use of medicinal plants is starting to play a secondary role. Ongoing globalisation is challenging the continuation of traditional medicinal health practices, as healthcare facilities have now improved and are geared more towards conventional medicine. Therefore, medical choices even in remote areas are progressively being influenced by these processes [74,75]. The tendency to acquire knowledge of plant medicines when the existence of an illness is identified is also observed in other ethnobotanical studies on cultural transmission [8,74]. Medicinal plants for self-medication in the countries of investigation are no longer essential and mainly offer people an alternative to conventional health practices if they have limited financial resources. The acquisition of plant knowledge is also often related to a personal quest and a person’s own interest and ability [26]. Lozada *et al.*[8] found that experienced traditional healers outside the family are important in knowledge transmission. Therefore it is assumed that knowledge of medicinal plants might increasingly become a domain of “expert knowledge”, more so in a societal context where people are no longer dependent upon the use of medicinal plants for healthcare. Different domains of knowledge may experience different secular changes. According to findings in a study among the Tsimané in the Bolivian Amazon, medicinal knowledge and wild food knowledge are experiencing a secular decline, especially among the generation born after 1970 [75].

## Conclusions

The analysis of network cards shows the transmission of knowledge about food and medicinal plants on an individual level. The knowledge networks visualise sources of knowledge that are important during a person’s lifetime, with additional information about gender, kinship and age. Its comparison allows patterns of knowledge transmission within a group and a certain domain to be identified.

Knowledge traditions of food and medicinal plants evolve historically within a specific social context. Food and medicinal plant knowledge is learned through language (oral and written), observation (real life, films, pictures) and being hands on, or a combination of these. During the process of transmission, the content of knowledge undergoes constant change and new interpretations. Tyrolean migrants and their descendants experienced different societal and environmental influences in the course of their migration history. New social networks, the strength of ties maintained with people in the country of origin and the age and life phase of the egos certainly had an influence on the quantity and kinds of sources of food and medicinal plant knowledge among egos. A lack of continuity in stable social relationships in a shared cultural environment may lead to different pathways of knowledge transmission or may even disrupt this process. The mode of transmission depends on the socio-economic conditions and the type of knowledge being transmitted. The findings in the present study indicate that vertical and horizontal transmission is the dominant path of knowledge transmission. In both domains, individual alters and relatives play a very important role in passing on knowledge of food and medicinal plants, as the proportion of knowledge transmitted within this group is highest and contact is most frequent.

In both domains, non-individual alters such as television, radio and the internet (oblique transmission) are mentioned as knowledge sources. These findings show that in more variable environments, *i.e.* in Australia where migrants are mainly dispersed in urban settings and have not formed communities, unlike migrants in Brazil and Peru, a shift in mode of knowledge transmission from vertical and horizontal towards the oblique path of transmission (many to many) can be observed. However, research [25,29] suggests that oblique transmission leads to uniformity within a social group and allows rapid cultural change. It is suggested that in a globalised world, the transmission of knowledge will progressively be influenced by the written word and new media. Social media such as the internet can be perceived as another variation of oblique transmission. However, Web 2.0 offers the opportunity for communities that are geographically remote but have similar migrational backgrounds for example to communicate and exchange their knowledge through this media. Since shifts in the transmission pathway may result in a fundamental change in the structure and content of knowledge [27,76] it is suggested that within the process of knowledge transmission, the role of mass media, *i.e.* books, the internet or television, should be investigated further to explore the parameters influencing these choices.

In future research it would be of interest to divide the investigation of knowledge transmission into different phases of an informant’s life. The investigation into knowledge transmission of temporal sequences of a person’s lifetime [77] will allow patterns of particular knowledge transmission pathways (vertical, horizontal or oblique) to be identified throughout a person’s lifetime. Observations about specific networks of food or medicinal plant knowledge over a longer timescale might reveal whether there are changes in the network structures and to what degree the composition and content of networks change over time. It is believed that the tools of social network analysis presented here make an important contribution to an improved understanding of the process of knowledge transmission since they refer to knowledge content and reveal its structures and characteristics.

## Competing interests

The authors declare that they have no competing interests.

## Authors’ contributions

RH, EK, HP, and CRV conceived and designed the research. RH, HP and EK carried out field research: HP in Australia, EK in Brazil and RH in Peru. RH and HP drafted the manuscript, and analysed and interpreted data. EK and CRV undertook a critical review of the manuscript for intellectual content. All authors read and approved the final manuscript.
